# SP3 and DEP1 Orchestrate Panicle Architecture by Jointly Regulating *APO2* Expression in Rice

**DOI:** 10.1002/advs.202508230

**Published:** 2025-09-03

**Authors:** Yuexin Liu, Niannian Chen, Xiaowei Fan, Xueqing Xia, Yilong Yao, Wen Huang, Jin Sun, Lintao Huang, Lei Wang, Hong Yu, Jiayang Li, Yongzhong Xing

**Affiliations:** ^1^ National Key Laboratory of Crop Genetic Improvement Hubei Hongshan Laboratory Huazhong Agricultural University Wuhan 430070 China; ^2^ Yazhouwan National Laboratory Sanya 572024 China

**Keywords:** *APO2* expression, DOF transcription factor, meristem activity, panicle architecture, protein interaction

## Abstract

Panicle architecture is largely determined by meristem activity. This previous study shows that DNA binding with one finger (Dof) transcription factor Short Panicle 3 (SP3) regulates panicle architecture. However, the molecular mechanisms of SP3 controlling panicle architecture remain largely unknown. Here, *SP3* is shown to enhance inflorescence meristem (IM) activity. Histological analysis shows that IM size rather than the timing of the meristem transition significantly reduces in *SP3* mutants. Several assays reveal that SP3 interacts with the C‐terminal cysteine‐rich domain of DENSE AND ERECT PANICLE1 (DEP1), a class C Gγ subunit, thereby regulating its plasma membrane–nucleus shuttle. SP3 directly binds to the *cis* element (A/T)AAAG located within the ‐561 to ‐517 bp region upstream of the *ABERRANT PANICLE ORGANIZATION2* (*APO2*) promoter and activates *APO2* expression, a positive regulator of panicle size. Genetic analysis indicates that *APO2* functions downstream of *SP3* to promote panicle branching. Additionally, loss of function of DEP1 increases *APO2* expression and spikelet density. Transcriptional activity assays show that the interaction between DEP1 and SP3 suppresses SP3‐mediated activation on *APO2*. Altogether, this study uncovers a transcriptional regulatory mechanism involving SP3 and DEP1 in controlling *APO2* expression, offering new insights into the genetic network underlying rice panicle development.

## Graphical abstract

The transcription factor SP3 enhances inflorescence meristem activity and interacts with the cysteine‐rich domain of DEP1. This interaction alters DEP1's subcellular localization between the plasma membrane and nucleus, and suppresses SP3‐mediated transcriptional activation of *APO2*. This study reveals a transcriptional regulatory mechanism involving SP3 and DEP1 in controlling *APO2* expression, providing new insights into rice panicle development.

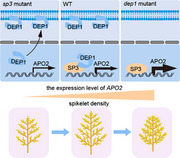

## 1 Introduction

Rice (*Oryza sativa* L.) grain yield is closely associated with inflorescence architecture, which consists of inflorescence axis, primary and secondary branches, as well as spikelets.^[^ ^1^ ^]^ Elucidating the genetic and molecular mechanisms underlying the control of basal structure is of great significance for breeding high yield varieties. Inflorescence architecture is largely determined by meristem identity and meristem activity. Some key genes determine the regulation of meristem fate, including initiation, maintenance, determinacy and dormancy, such as *LAX PANICLE1* (*LAX1*), *LAX2*, *ABERRANT SPIKELET AND PANICLE1* (*ASP1*) and *FRIZZY PANICLE* (*FZP*).^[^ ^2^, ^3^, ^4^, ^5^ ^]^ Some crucial genes involved in cytokinin (CK) synthesis affect the activity of meristematic tissue and cell division resulting in changed panicle structure. The accumulation of active CKs in young panicles enhances meristem activity and grain yield. For example, downregulated expression of cytokinin oxidase/dehydrogenase *GRAIN NUMBER1a* (*Gn1a*)/*OsCKX2* and *GY3* encoding phosphohydrolase of cytokinin nucleotide enhances the number of spikelets and grain yield mainly by increasing secondary branches.^[^ ^6^, ^7^ ^]^

ABERRANT PANICLE ORGANIZATION 2 (APO2)/RFL is the ortholog of *Arabidopsis* LEAFY (LFY) in rice.^[^ ^8^ ^]^ The loss‐of‐function mutant *apo2* produces small panicles with fewer primary and secondary branches.^[^ ^9^ ^]^ Further studies revealed that *APO2* suppresses the transition from inflorescence meristem(IM) to floral meristem(FM) and positively controls meristem cell proliferation after the reproductive transition.^[^ ^9^, ^10^ ^]^ *Dense and Erect Panicle1* (*DEP1*) encodes the γ‐subunit of a heterotrimeric G‐protein complex that regulates meristem activity, resulting in an increased grain number.^[^ ^11^ ^]^ DEP1 mediates differential regulation of downstream genes through diverse protein–protein interactions. For example, DEP1 interacts with MADS transcription factors (TFs) and enhances OsMADS1 transcriptional activity.^[^ ^12^ ^]^ In addition, DEP1 also functions in nitrogen use efficiency and grain size by interacting with Gα (RGA1) and Gβ (RGB1) subunits at the plasma membrane or in the nucleus.^[^ ^13^, ^14^ ^]^

The DNA binding with one finger (Dof) family is a plant‐specific TF family sharing a highly conserved single Cys2/Cys2 zinc finger DNA‐binding domain.^[^ ^15^ ^]^ Dof TFs are involved in regulatory networks controlling diverse developmental processes and phytohormone signaling pathways, as well as plant responses to abiotic stresses. They regulate the expression of target genes via recognizing (A/T)AAAG sequence or its inverse sequence CTTT(A/T) in promoters.^[^ ^16^, ^17^, ^18^, ^19^ ^]^ In rice, 30 Dof TFs have been identified.^[^ ^15^ ^]^ Among them, OsDof12 regulates plant architecture by suppressing brassinosteroid (BR) signaling.^[^ ^20^, ^21^ ^]^ It also interacts with GIGANTEA (OsGI) and attenuates its transactivation activity on *OsPAL4*, which encodes phenylalanine ammonia‐lyase 4, a key enzyme in the phenylpropanoid pathway.^[^ ^20^ ^]^ OsDof4 modulates flowering time by reducing photoperiod sensitivity.^[^ ^22^ ^]^ OsDOF18 improves head rice yield by activating *Wx^b^* and *ALK^b^* expression to form less amylose and more amylopectin, in addition to mediating ammonium transport and nitrogen distribution.^[^ ^23^, ^24^ ^]^ OsDOF24 suppresses the induction of leaf senescence in jasmonate biosynthetic pathway.^[^ ^25^ ^]^ In Arabidopsis, *COGWHEEL1*, the homologous genes of *OsDof5*, enhances photosynthetic capacity and starch accumulation in rosette leaves.^[^ ^26^ ^]^ The mechanism of Dof15 regulating primary root elongation and tiller development has been reported.^[^ ^27^, ^28^ ^]^ Despite these advances, the molecular mechanisms by which Dof TFs integrate developmental and signaling pathways to coordinate rice panicle development remain poorly understood.

In our previous research, *Small Panicle 3* (*SP3*) encoding Dof15 controls inflorescence architecture by regulating the content of cytokinins,^[^ ^29^ ^]^ but the mechanism governing inflorescence architecture remains unknown. Here, we demonstrated that *SP3* controls panicle length and grain number by regulating meristem activity. SP3 interacts with DEP1 and modulates the subcellular localization of DEP1. SP3 targets the promoters of *APO2* and upregulates *APO2* expression together with DEP1. These findings reveal a SP3‐DEP1‐APO2 molecular module controlling panicle length and grain number in rice.

## 2 Results

### 2.1 *SP3* Knockout Mutant Has Short Panicles with a Small Number of Spikelets

**Figure 1:**
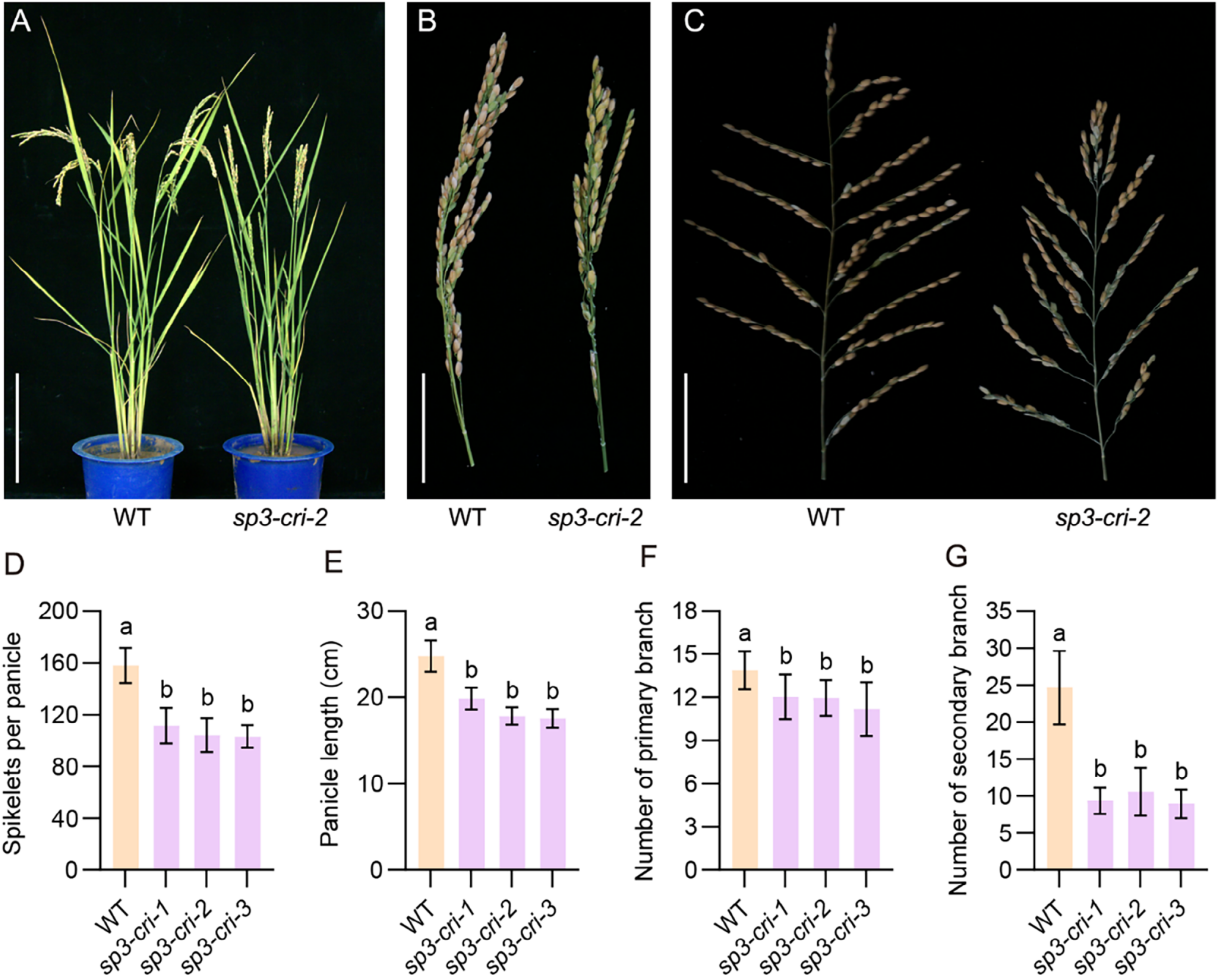
Comparison of panicle traits between the wild type and *SP3* knockout mutant. Comparison of the A) whole plants, B) panicles, and C) panicle architecture at the maturation stage between WT and *sp3‐cri‐2*. Scale bars, A) 20 cm, B,C) 10 cm (B.Comparison of D) spikelet per panicle, E) panicle length (cm), F) primary branch number (and G) secondary branch number between WT and *SP3* knockout mutants. Values are given as mean ± SD (*n* = 10). The different lowercase letters above the histogram indicate significant differences by Duncan's test (*P* < 0.05).

*SP3* was identified from a short panicle mutant in which T‐DNA was inserted in the promoter of *Dof15*.^[^ ^29^ ^]^ The *sp3* mutant had smaller panicles and fewer branches than wildtype (WT) plants. Three *Dof15* edited lines were generated in the background of Zhonghua 11 by using the CRISPR/Cas9 system. Comparative analysis of gene and amino acid sequences showed that frameshift mutations in *sp3‐cri‐1* and *sp3‐cri‐2* disrupt protein function, whereas *sp3‐cri‐3* exhibits premature translation termination (Figure S1 and Table S1, Supporting Information). Compared with the WT plant, *sp3‐cri* mutants had a reduced plant height and shortened panicles (**Figure** **1**A–C). Duncan's test revealed that panicle length and both the numbers of primary and secondary branches were significantly reduced, leading to a dramatic reduction of spikelets per panicle (Figure 1D–G). Transgenic lines overexpressing *SP3* (*SP3‐OX‐1* and *SP3‐OX‐2*) were generated (Figure S2, Supporting Information). The expression of *SP3* was markedly increased in both *SP3‐OX‐1* and *SP3‐OX‐2* plants compared with WT (Figure S2, Supporting Information). However, no significant differences in panicle‐related traits were observed between *SP3*‐overexpressing plants and WT (Figure S2, Supporting Information).

### 2.2 *SP3* Enhances Inflorescence Meristem Activity

**Figure 2:**
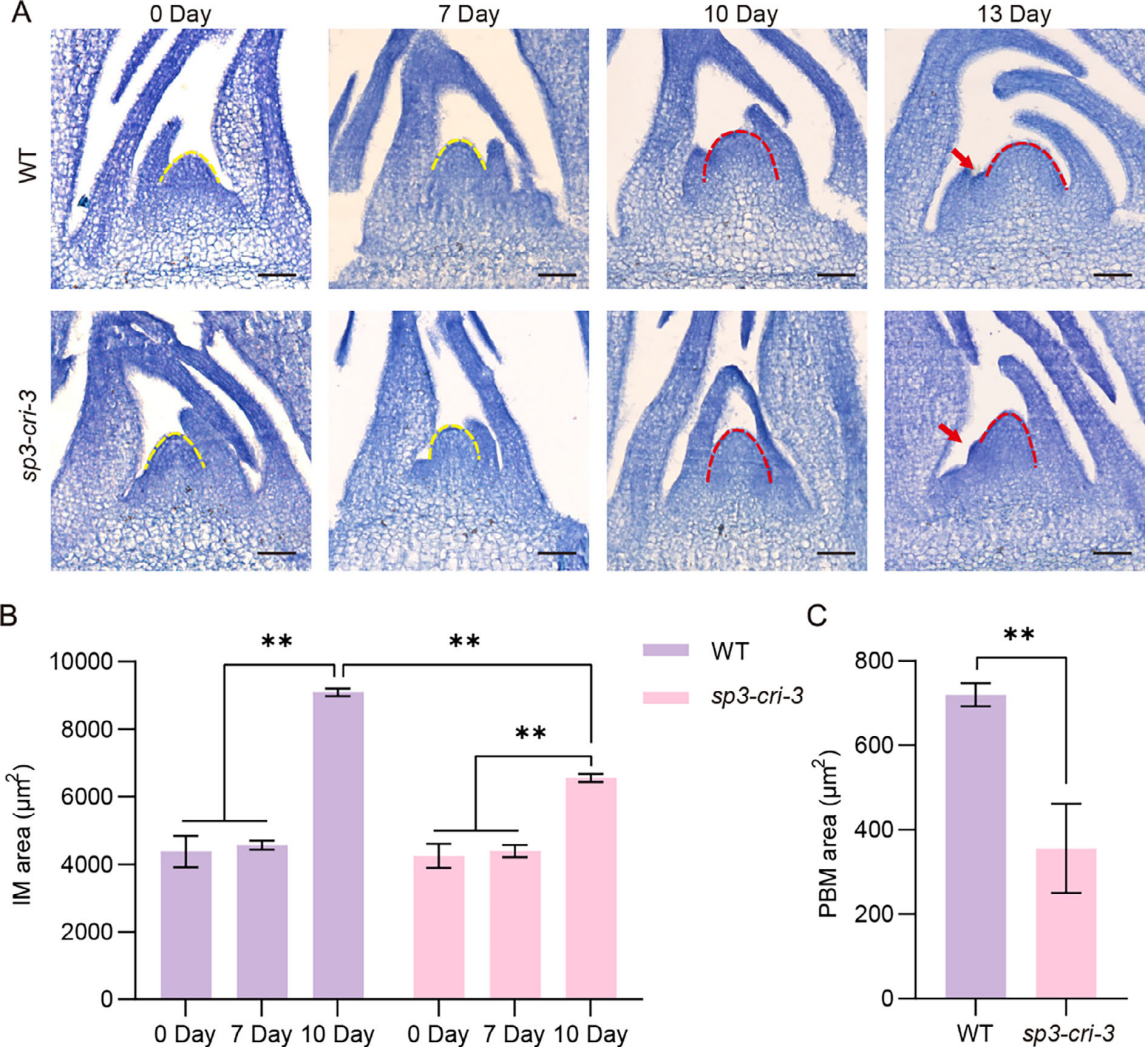
Comparison of SAMs and IMs between WT and *sp3‐cri‐3* plants. A) Meristems in the WT and *sp3‐cri‐3* at 0 and 7, 10, and 13 d after SD condition. The yellow dotted line outlines the meristem region during the vegetative stage and the red dotted line outlines the IM after the reproductive transition. Red arrows indicate PBM. Scale bars = 50 µm. The areas of B) IM and C) 13 day old PBM in WT and *sp3‐cri‐3* mutant measured by ImageJ. The data represent the mean ± SD (n = 3). *P* values were calculated by two‐sided paired Student's *t*‐test, **P < 0.01.

To determine if *SP3* is involved in the control of meristem activity, we dynamically compared shoot apical meristem development in the *sp3‐cri‐3* mutant with that in WT. After 30 days of growth under long‐day (LD) conditions, plants were transferred to short‐day (SD) conditions. Samples were collected on days 0, 7, 10, and 13 during the SD period. The size and shape of shoot apical meristem (SAM) had no significant difference between the *sp3‐cri‐3* mutant and WT within the first seven days (Days 0 and 7) (**Figure** **2**A,B). The SAM enlargement was observed on Day 10 and the primary branch meristems (PBMs) were simultaneously observed on Day 13 in WT and *sp3‐cri‐3* mutant, suggesting that the timing of the meristem transition was not affected in the *sp3‐cri‐3* mutant (Figure 2). Moreover, the size of IM and PBMs were significantly smaller in the *sp3‐cri‐3* mutant compared with the WT (Figure 2B,C). This result implies that *SP3* does not alter the reproductive transition but positively regulates activity of panicle meristems.

*KNOX* genes are required for meristem maintenance, and their loss‐of‐function mutations lead to abnormal inflorescence development.^[^ ^30^ ^]^ Therefore, we measured the expression levels of several *KNOX* genes such as *OSH1*, *OSH3*, *OSH6* and *OSH15* in young panicles (≈0.2 cm). The expression levels of these four genes were significantly downregulated in the mutants (Figure S3A–D, Supporting Information). We also examined the expression of several other genes involved in meristem activity and panicle development in rice. *OsWUSCHEL*, encoding a positive regulator of IM development, and several genes associated with panicle development including *IPA1*, *ASP1*, and *RCN2*, were downregulated in the *sp3‐cri* mutant (Figure S3E–H, Supporting Information). Taken together, SP3 may enhance rice IM activity, at least in part, by influencing the expression of genes encoding positive regulators of IM development, thereby contributing to the formation of long panicles with multiple branches.

### 2.3 SP3 Interacts with DEP1

**Figure 3:**
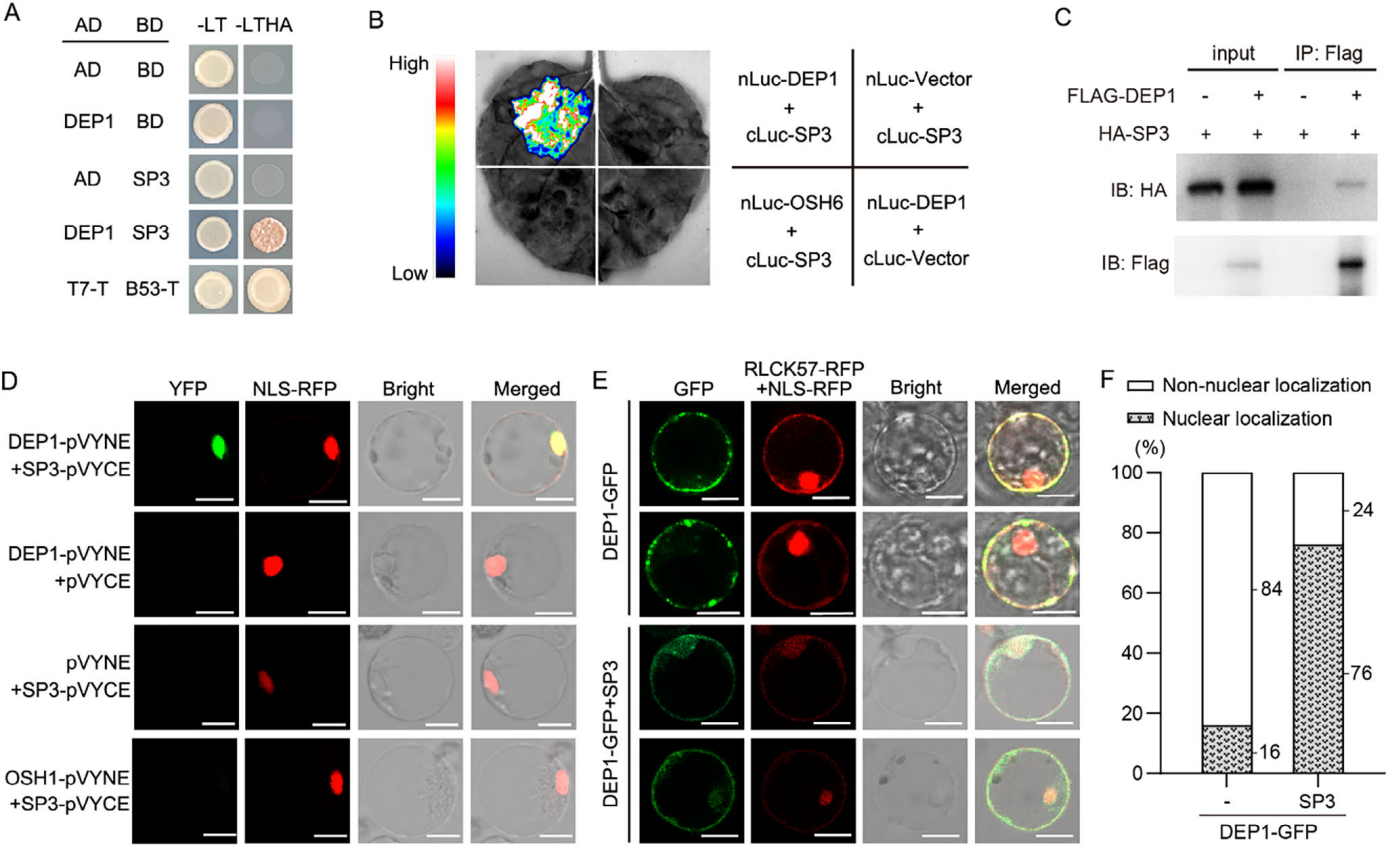
SP3 interacts with DEP1 and modulates DEP1 plasma membrane–nuclear shuttling. A) A yeast two‐hybrid assay for the interaction between SP3 and DEP1. Co‐expression of pGADT7‐T/pGBKT7‐53 was used as positive control; co‐expression of AD/BD was used as negative control; co‐expression of DEP1‐AD/BD and AD/SP3‐BD were used as self‐activation detection. The screening medium are SD‐Trp‐Leu His‐Ade and SD‐Trp‐Leu. B) Luciferase complementation imaging assays showing the interaction between SP3 and DEP1. cLUC‐SP3 was co‐transformed into tobacco leaves along with either nLUC‐DEP1 or nLUC‐Vector. nLUC‐OSH6 was used as the negative controls. Colored scale bar indicates the luminescence intensity in CPS. C) Co‐IP assays demonstrate the in vivo interaction between SP3 and DEP1. Pro35S:FLAG‐DEP1 and pro35S:HA‐SP3 fusions were co‐expressed in rice protoplast. Proteins were extracted (Input) and immunoprecipitated (IP) with FLAG beads. Western blotting was performed using anti‐FLAG and anti‐HA antibodies. D) BiFC assays show SP3‐DEP1 interaction in rice protoplasts. OSH‐pVYNE was used as the negative controls. Scale bars, 10 µm. E) Analysis of the subcellular localization of DEP1‐GFP with or without SP3 using rice protoplast transient expression. RLCK57‐RFP serves as a plasma membrane marker. NLS‐RFP serves as a nuclear marker. Scale bars, 10 µm. F)Statistical analysis of the subcellular localization shown in (E). At least 30 protoplasts were counted for each combination. The experiment was performed with 2 technical replicates.

To identify SP3 interacting proteins, a young panicle cDNA library was screened using the yeast two‐hybrid (Y2H) system with full‐length SP3 as bait after confirming the absence of self‐activation in the full‐length SP3 protein (**Figure** **3**A). Several non‐frameshift candidate genes were identified, including LOC_Os11g06760, LOC_Os03g60600, LOC_Os05g02780, and LOC_Os09g26999. Among them, LOC_Os09g26999 encodes DEP1, classified as a class C Gγ subunit. The interaction between DEP1 and SP3 was further confirmed by pairwise Y2H assays (Figure 3A). Subsequent assays demonstrated that DEP1 interacts with the zinc finger DNA‐binding domains of SP3 (Figure S4, Supporting Information). To further confirm this interaction, firefly luciferase complementation imaging (LCI) assays were performed by co‐expressing nLUC‐DEP1 and cLUC‐SP3 transiently in *N. benthamiana* leaves. Strong signals were observed in leaves in which nLUC‐DEP1 and cLUC‐SP3 were co‐expressed. Whereas no detectable signals were observed for other combinations (Figure 3B). To assess the interaction between SP3 and DEP1 in vivo, FLAG‐DEP1 and HA‐SP3 proteins were expressed transiently in rice protoplasts and then purified and subjected to co‐immunoprecipitation (Co‐IP) assays. The results showed that HA‐SP3 was co‐precipitated with FLAG‐DEP1, but not with the negative control (Figure 3C). Subsequently, the in vivo interaction between SP3 and DEP1 in the nucleus was confirmed by bimolecular fluorescence complementation (BiFC) experiments in rice protoplasts (Figure 3D).

### 2.4 SP3 Alters the Subcellular Localization of DEP1

The localization of SP3 as a TF in the nucleus has been reported.^[^ ^29^ ^]^ Whereas there were some controversies over subcellular localization of DEP1. DEP1 was previously reported to localize exclusively on the cell membrane,^[^ ^14^ ^]^ both on the cell membrane and nucleus,^[^ ^13^ ^]^ or solely in the nucleus.^[^ ^31^ ^]^ Here, BiFC assays demonstrated that SP3 and DEP1 interact within the cell nucleus (Figure 3D). To clarify the subcellular localization of DEP1, *Pro35s:DEP1‐GFP* was transiently expressed in rice protoplasts. In the absence of SP3, DEP1‐GFP is predominantly co‐localized with the plasma membrane marker RLCK57‐RFP, indicating that DEP1 is mainly localized at the plasma membrane (Figure 3E). To test whether the interaction SP3‐DEP1 affects the subcellular localization of DEP1, *Pro35s:DEP1‐GFP* and *Pro35s:SP3‐HA* were transiently co‐expressed in rice protoplasts. Under these conditions, DEP1‐GFP signals were observed to co‐localize with both RLCK57‐RFP and the nuclear marker NLS‐RFP (Figure 3E). Quantitative analysis revealed that 16% of DEP1‐GFP signals were localized to the nucleus in the absence of SP3, whereas this proportion increased significantly to 76% upon co‐expression with SP3 (Figure 3F). The results indicate that SP3 alters the localization of DEP1 and modulates DEP1 cytoplasmic–nuclear shuttling.

### 2.5 Cysteine‐Rich Domain Is Essential for Protein Interaction

DEP1 is considered a plant‐specific G protein γ subunit. According to predictions from the SMART database, a Gγ‐like domain is located between amino acids 24 and 107 of DEP1.^[^ ^32^ ^]^ Additionally, the NCBI database predicts a VESA1 (variant erythrocyte surface antigen‐1) domain between amino acids 237 and 314. Moreover, DEP1 contains a cysteine‐rich domain at its C‐terminal region. This domain is the primary site where numerous natural variations are concentrated, and it also mediates the interaction between DEP1 and TFs.^[^ ^12^, ^14^, ^31^, ^33^, ^34^ ^]^ Y2H analysis revealed that SP3 does not interact with the truncated DEP1 lacking the C‐terminal cysteine‐rich domain but does interact with the truncated form retaining this domain, indicating that the C‐terminal cysteine‐rich domain of DEP1 is essential for its interaction with SP3 (Figure S5B, Supporting Information). LCI assays showed that SP3 interacts with C‐terminal cysteine‐rich domain (DEP1^Cys‐rich2^) of DEP1 instead of DEP1^Gγ‐like^, DEP1^Cys‐rich1^ and DEP1^VESA1^ (Figure S5C, Supporting Information). These results further confirm the interaction between SP3 and the C‐terminal cysteine‐rich domain of DEP1.

### 2.6 The C‐Terminus Cysteine‐Rich Domain of DEP1 Has Strong Membrane Localization

To determine the role of the C‐terminus cysteine‐rich domain in its subcellular localization, the truncated DEP1 proteins mentioned above were individually fused to GFP at either the N‐ or C‐terminus. The subcellular localization of each fusion protein was then examined to assess the contribution of different domains. N‐terminal and C‐terminal GFP fusion proteins of DEP1^Gγ‐like^ and DEP1^Gγ‐like + Cys‐rich1^ were localized to both the plasma membrane and the nucleus (Figure S6, Supporting Information). N‐terminal and C‐terminal GFP fusions of DEP1^VESA1^ and DEP1^Cys‐rich2^ were localized to the plasma membrane, whereas the fusion proteins DEP1^VESA1+Cys‐rich2^ and DEP1^Cys‐rich1+VESA1+Cys‐rich2^ were localized to both the plasma membrane and the endoplasmic reticulum (Figure S6, Supporting Information). These results suggest that the Gγ‐like domain of DEP1 contains a nuclear localization signal, whereas its C‐terminal cysteine‐rich domain harbors a strong membrane localization signal.

### 2.7 SP3 Upregulates the Expression of *APO2* by Binding to Its Promoter

**Figure 4:**
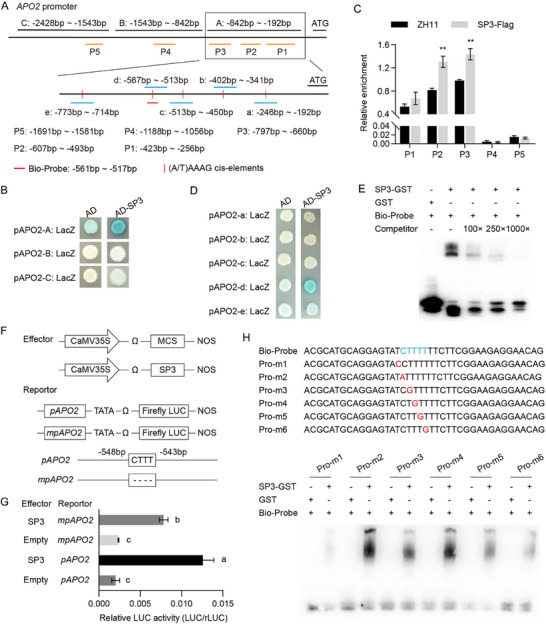
Assay of SP3 binding to the promoter of *APO2*. A) Illustration of fragments in the 2.5‐kb *APO2* promoter region. Fragments A to C indicate segmentation of *APO2* promoters. Fragments P1 to P5 were used for designing the primers used in the ChIP‐PCR experiments. Fragments a‐e represent five (A/T)AAAG cis‐element enrichment regions in the yeast one‐hybrid experiment. B) Yeast one‐hybrid assay verified the interaction between SP3 and fragment A. pAPO2‐A:LacZ, pAPO2‐B:LacZ, and pAPO2‐C:LacZ represent the three segments of the *APO2* promoters A, B, and C that were connected to the pLacZi2μ vector, X‐gal was added to measure the reporter gene LacZ. C) ChIP‐qPCR results showing that SP3 binds to the promoter of *APO2* in vivo. Chromatin isolated from 2 mm young panicles of UBI:SP3‐FLAG transgenic plants and wild‐type were immunoprecipitated with the anti‐FLAG antibody. The data represent the mean ± SD (*n* = 3). *P* values were calculated by two‐tail paired Student's *t*‐test, **P < 0.01. (D) Yeast one‐hybrid assay for verification of the interaction between SP3 and fragment d in the *APO2* promoter. pAPO2‐a:LacZ, pAPO2‐b:LacZ, pAPO2‐c:LacZ, pAPO2‐d:LacZ, and pAPO2‐e:LacZ representing the five segments of the *APO2* promoters were connected to the pLacZi2μ vector. E) EMSA assays. Competition for SP3‐GST binding was performed with 100×, 250× and 1000× unlabeled probes containing the cis‐element (A/T)AAAG. GST protein served as negative control. F) Schematic diagram of effector constructs, and reporter constructs used in the transient LUC assay. The full‐length CDS of SP3 were driven by the CaMV35S promoter. The reporter gene LUC was driven by the *APO2* promoter. The fragment ‐2303 to ‐93 bp upstream of *APO2* promoter and its mutated derivatives were inserted in the region before TATA box. G) Relative LUC activity of combinations of the different effectors and reporters described above. Relative LUC activity is represented by the ratio of signal values of firefly LUC to that of *Renilla* LUC (REN). Data are shown as the mean ± SD of three independent transformants. Different lowercase letters indicate significant differences among the different combinations between reporters and effectors by Duncan's multiple range tests (*P*<0.05). H) EMSA confirmed that SP3 bound to the cis‐element (A/T)AAAG. Stepwise single‐nucleotide mutation probes were generated to identify the core binding element. The red nucleotide indicates the specific base position of the mutant probe.

Our previous study revealed that the expression level of *APO2* was significantly reduced in approximate 2 mm young panicles of the *sp3* mutant^12^, ^14^, ^29^ ^]^ Multiple core motifs (A/T)AAAG *cis‐*elements recognized by DOF TFs were identified in a 2‐kb region upstream of *APO2* translation start site, which suggested the possibility of potential direct interactions between SP3 and the promoter of *APO2*. Therefore, the 2 kb fragment was divided into three fragments A, B and C (**Figure** **4**A). A yeast one‐hybrid (Y1H) assay demonstrated that SP3 interacted with fragment A, which covered the *APO2* promoter region from ‐842 bp to ‐192 bp (Figure 4B). Subsequently, chromatin immunoprecipitation‐qPCR (ChIP‐qPCR) analysis demonstrated SP3 binding to fragments P2 (‐607 to ‐493 bp) and P3 (‐797 to ‐660 bp) (Figure 4C), which further confirmed the interaction between SP3 and fragment A. To identify the fine binding sites, fragment A was further processed into five fragments a, b, c, d and e, each containing one (A/T)AAAG *cis* element (Figure 4A). The Y1H assays showed that SP3 directly bound to fragment d, which covered the 55 bp region from ‐567 bp to ‐513 bp containing a *cis* element of (A/T)AAAG (Figure 4D). To determine the precise binding site, an electrophoretic mobility shift assay (EMSA) probe was designed based on fragment d (Bio‐Probe fragment: from ‐561 bp to ‐517 bp). The EMSA showed that the binding motif was located in the Bio‐Probe fragment (Figure 4E). Together, SP3 binds to (A/T)AAAG *cis* element located in the region of Bio‐Probe fragment (‐561 bp to ‐517 bp) in the promoter of *APO2*.

Transient expression assays were performed to confirm the upregulation of SP3 to *APO2* expression in rice protoplasts. The 2‐kb fragment upstream of the *APO2* transcription start site (TSS) and derivative with deletion of the *cis*‐element (A/T)AAAG located in the region of Bio‐Probe fragment were integrated with a LUC reporter gene to construct reporter plasmids (Figure 4F). The *SP3* coding sequence driven by the promoter CaMV35S was used as effectors (Figure 4F). Compared with the negative control (No effector), the SP3 effectors exhibited a significant activation effect on the *pAPO2* reporter (Figure 4G). In addition, the deletion of the *cis* element (A/T)AAAG (*mpAPO2*) weakened these active effects because the relative LUC activities were significantly decreased compared with the levels of the *pAPO2* reporter (Figure 4G). Moreover, EMSA results showed that the mutated probe retaining the intact (A/T)AAAG motif could compete with the labeled wild‐type probe, whereas the mutated probes each with a disrupted motif failed to compete (Figure 4H). Thus, the activation of *APO2* by SP3 requires the *cis* element (A/T)AAAG.

### 2.8 SP3 Regulates Panicle Architecture by Activating *APO2*

**Figure 5:**
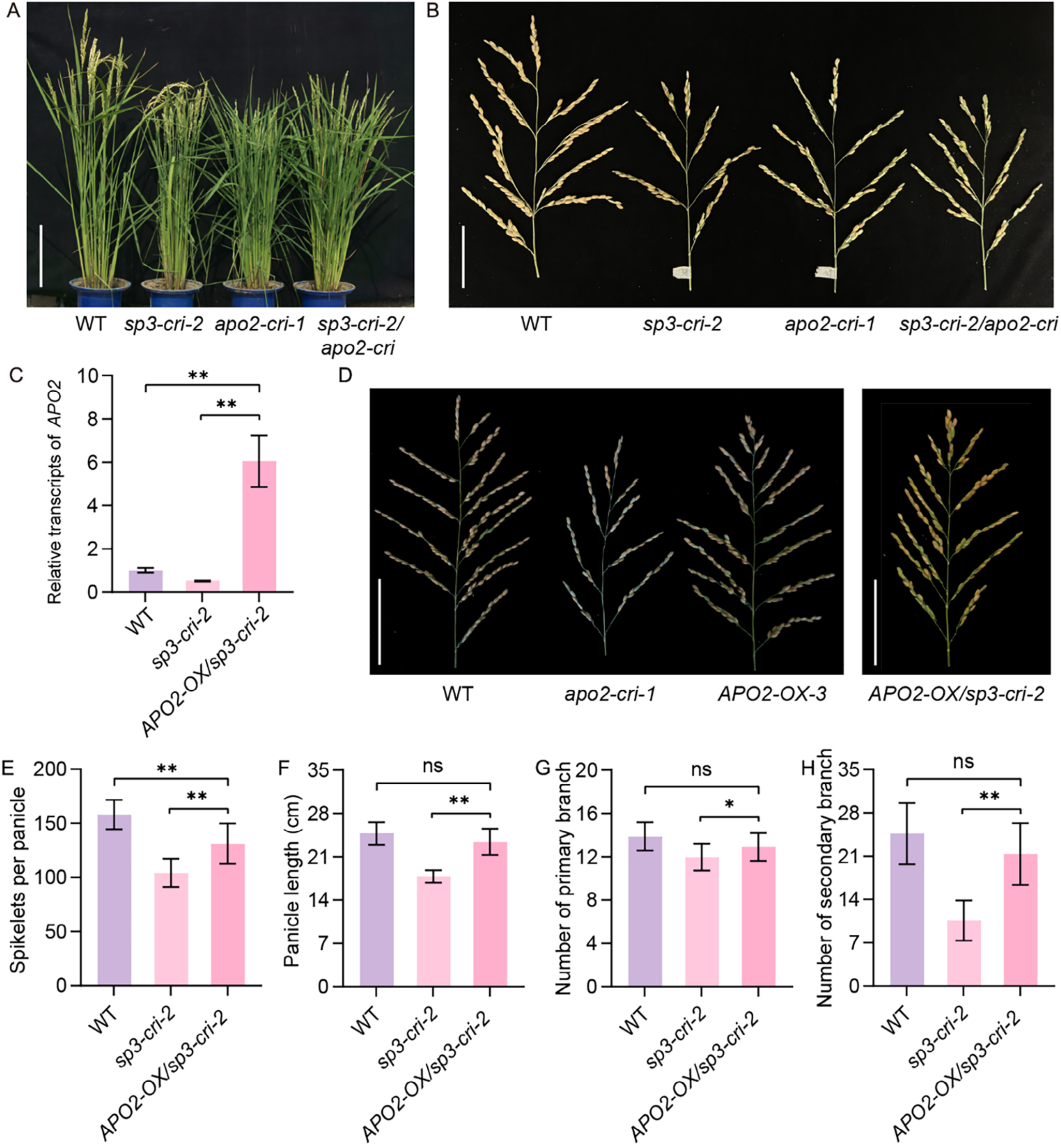
Overexpression of *APO2* rescued *SP3* mutant phenotype. Comparisons of A) the whole plants and B) panicle architecture at the maturation stage between WT, *sp3‐cri‐2*, *apo2‐cri‐1* and *sp3‐cri‐2/apo2‐cri*. Scale bars, A) 20 cm, B) 8 cm. C) Relative *APO2* expression levels in WT, *APO2*‐overexpressing plants and *APO2‐OX/ sp3‐cri‐2*. The data represent the mean ± SD (n = 3). *P* values were calculated by two‐sided paired Student's *t*‐test, *P < 0.05, **P < 0.01. D) Comparisons of the panicle architecture among WT, *apo2‐cri‐1, APO2‐OX‐1* and *APO2‐OX/sp3‐cri‐2*. Scale bars, 10 cm. Comparison of E) spikelet per panicle, F) panicle length, G) number of primary branches and H) secondary branches among WT, *sp3‐cri‐2* and *APO2‐OX/sp3‐cri‐2* (n = 10 panicles). The data represents the mean ± SD (n = 10). *P* values were calculated by two‐sided paired Student's *t*‐test, *P < 0.05, **P < 0.01.

To clarify the regulatory relationship between SP3 and APO2 in panicle architecture, three *APO2* homozygous knockout lines were generated using CRISPR/Cas9 technology. (Figure S7A, Supporting Information). Compared with WT plant, the *apo2‐cri* mutants had a reduced plant height and shortened panicles, similar to *sp3‐cri* mutants (Figure S7B,C, Supporting Information). Moreover, spikelet per panicle, panicle length and both the numbers of primary and secondary branches were significantly reduced (Figure S7D–G, Supporting Information). The double mutants (*sp3‐cri‐2/apo2‐cri*) also exhibited a reduced plant height and shortened panicles, similar to *sp3‐cri* mutants and *apo2‐cri* mutants (Figure S8, Supporting Information and **Figure** **5**A,B). Therefore, both *SP3* and *APO2* function in the same pathway regulating panicle architecture.

*APO2*‐overexpressing plants (*APO2‐OX‐3*), and the double mutants overexpressing *APO2* in the *SP3* knockout background (*APO2‐OX/sp3‐cri‐2*) were generated to demonstrate whether SP3 regulates panicle architecture by activating *APO2* (Figure 5C and Figure S9, Supporting Information). The expression of *APO2* sharply elevated in the *APO2*‐OX‐3 and *APO2‐OX/sp3‐cri‐2* compared with WT (Figure 5D and Figure S9B, Supporting Information). There is no significant difference in panicle traits between *APO2*‐OX‐3 and WT (Figure S9C–F, Supporting Information). But *APO2‐OX/sp3‐cri‐2* lines had significantly increased spikelet per panicle, panicle length and both the numbers of primary and secondary branches as compared with *sp3‐cri‐2*, which was similar to WT (Figure 5D–H). Taken together, overexpressing *APO2* well rescued the reduced branches in the *SP3* knockout mutants.

### 2.9 DEP1 Inhibits the Binding of SP3 to Its Target *APO2*

**Figure 6:**
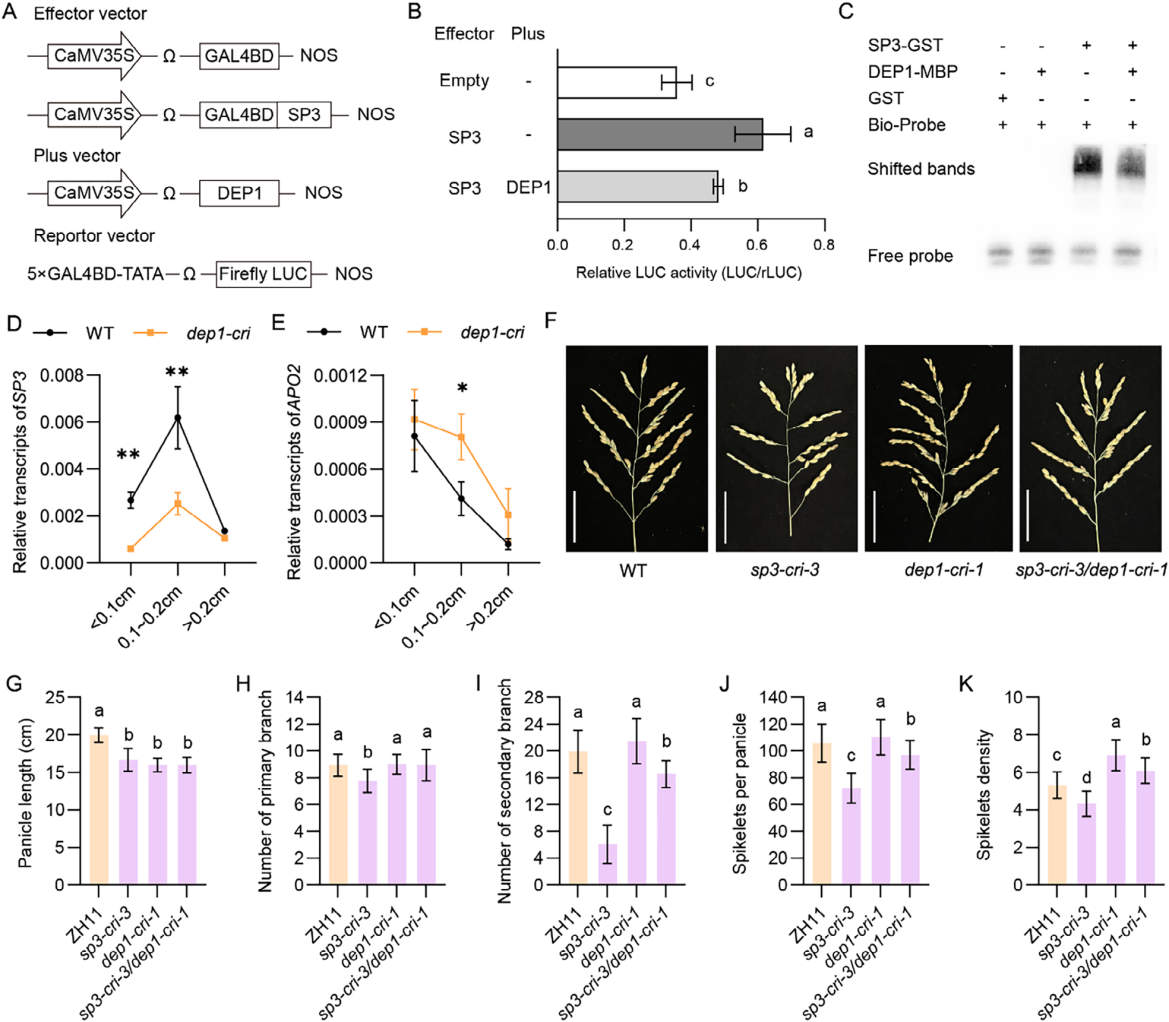
DEP1 prevents SP3 from binding to its targets and regulates grain number. A) Schematic diagram of effector constructs, and reporter constructs used in the transient LUC assay. SP3 were fused to the GAL4‐binding domain (GAL4BD). The relative activity of firefly luciferase (LUC) under control of the GAL4‐binding was measured. B) Determination of the transcriptional activity of SP3 in protoplasts. Data are shown as the mean ± SD of three independent transformants. Different lowercase letters indicate significant differences among the combinations between reporters and effectors by Duncan's multiple range tests (*P*<0.05). C) EMSA shows that DEP1 and SP3 suppress the binding activity of SP3 to the *APO2* promoter. GST protein served as negative control. D,E) Comparison of *SP3* and *APO2* expression in young panicles at different lengths stages by RT‐qPCR between WT and *dep1‐cri*. The data represent the mean ± SD (*n* = 3). *P* values were calculated by two‐sided paired Student's *t*‐test, *P < 0.05 and **P < 0.01. (F) Performances of panicle architecture at maturation stage of WT, *sp3‐cri‐3*, *dep1‐cri‐1* and hybrids of *sp3‐cri‐3* and *dep1‐cri‐1*. Scale bars, 5 cm. Statistical comparison of G) panicle length, H) primary branch number, I) secondary branch number, J) grain number and K) spikelet density among the WT, *sp3‐cri‐3*, *dep1‐cri‐1* and hybrids of *sp3‐cri‐3* and *dep1‐cri‐1*. Values are given as mean ± SD (*n* = 10). The different lowercase letters above the histogram indicate significant differences by Duncan's test (*P* < 0.05).

The transient transcriptional activity assays showed that SP3 transactivation activity was substantially weakened when SP3 interacted with DEP1 (**Figure** **6**A,B). LUC activity driven by the *APO2* promoter was induced by SP3, while it was substantially inhibited when SP3 interacted with DEP1 (Figure S10A,B, Supporting Information). These results suggest that the SP3–DEP1 interaction inhibits the transactivation activity of SP3. In addition, similar LUC activity driven by *mpAPO2* was observed between the cases with the effector DEP1 and without the effector DEP1 (Figure S10C, Supporting Information), indicating the importance of cis element of *APO2* in regulating its transcriptional expression. EMSA showed that it is SP3‐GST rather than DEP1‐MBP fusion proteins directly bound to Bio‐probe and the presence of DEP1 inhibited the binding activity of SP3 to the *APO2* promoter (Figure 6C).

Secondary branch meristematic (SPM) is dramatically developed in the 0.1–0.2 cm young panicles.^[^ ^35^ ^]^ In WT, *SP3* transcription gradually increases in the 0.1–0.2 cm young panicles and then decreases in more than 0.2 cm young panicles, while the expression level of *APO2* continues to decrease (Figure 6D,E). In *dep1‐cri*, the expression level of *SP3* in the 0.1–0.2 cm young panicles remains higher than in those larger than 0.2 cm, but its expression level is significantly lower than in WT (Figure 6D). However, in the 0.1–0.2 cm young panicles of *dep1‐cri*, the expression level of *APO2* is significantly higher than in WT (Figure 6E). Therefore, DEP1 functions as a cofactor of SP3 in downregulating *APO2*, and its deficiency leads to the upregulation of *APO2*, which in turn increases the number of branches and spikelet density in *dep1‐cri*.

When compared with WT, the *dep1* knockout mutant had a significant decrease in panicle length, but a significant increase in spikelet density (Figure S11, Supporting Information), which is similar to NIL‐*dep1*.^[^ ^11^ ^]^ Both the double mutants and the two single mutants exhibited a similar change in panicle architecture characterized with shortened panicle length, but the numbers of secondary branches, spikelets per panicle and spikelet density in the double mutants were less than that in *dep1‐cri‐1* but more than in *sp3‐cri‐3* (Figure 6F–K). In addition, the expression levels of *OsWUS*, *IPA1* and *RCN2* controlling meristem activity were upregulated in young panicles of *dep1‐cri*, opposite to that in *sp3‐cri* (Figure S3, Supporting Information). Taken together, *dep1* knockout mutant enhances meristematic activity through promoting APO2 and other genes.

## 3 Discussion

### 3.1 The SP3‐DEP1‐APO2 Module Regulates Panicle Architecture

In this study, we identified that the *sp3* knockout mutant resulted in shorter panicles with reduced numbers of branches and spikelets (Figure 1A–G). The tissue slicing experiment verified the reduction in IM activity, which causes the varied panicle architecture in *sp3‐cri* mutant (Figure 2). This implies that SP3 controls inflorescence length and panicle architecture by regulating IM activity, which is highly similar to *APO2* (Figure 2). *APO2* regulates the proliferation of meristematic cells after the reproductive transition and mediates the transition from FM to spikelet meristem, exhibiting pleiotropic effects.^[^ ^9^, ^10^ ^]^ APO2 interacts with APO1 to jointly regulate the transition from IM to FM, and it modulates its own stability by interacting with the E3 ubiquitin ligase LARGE2.^[^ ^36^ ^]^ However, the transcriptional regulatory mechanism of *APO2* remains unclear. The previous study revealed that *SP3* and *APO2* have the same expression pattern in young panicles.^[^ ^29^ ^]^ Here, *APO2* expression was significantly downregulated in the *sp3* mutant (Figure 5A). The 2 kb region upstream of *APO2* TSS possesses 15 (A/T)AAAG *cis‐*elements, the core binding motifs of Dof TFs.^[^ ^19^ ^]^ Biochemical experiments showed that SP3 binds to the *cis‐*element (A/T)AAAG locating in ‐561 to ‐517 bp in the *APO2* promoter and then activates the expression of *APO2* (Figure 4D–H). The double mutant *sp3‐cri‐2/apo2‐cri* exhibits similar panicle length and branch numbers to the single mutants *apo2‐cri* and *sp3‐cri‐2* (Figure 5B), and overexpression of *APO2* partially restored the panicle length and the number of branches in *sp3* mutant, indicating that SP3 regulates panicle development through activating *APO2* (Figure 5).

DEP1 interacts with the conserved keratin‐like domain of MADS TFs, such as OsMADS1, enhancing their transcriptional activity and facilitating the co‐transcriptional activation of shared target genes.^[^ ^12^ ^]^ In this study, DEP1 interacts with the DOF‐domain of SP3 and inhibits its transcriptional activation activity of SP3, thereby further downregulating *APO2* expression. The reduced *SP3* expression and increased *APO2* expression in the inflorescences of *DEP1* knockout mutants suggests a repressive role of DEP1 in SP3‐mediated transcriptional activation of *APO2*. This antagonistic regulation of *APO2* by SP3 and DEP1 provides a plausible explanation for the reduced secondary branch number in *SP3* knockout mutants and the increased secondary branch number in *DEP1* knockout mutants. However, we do not have additional evidence to explain the cause of the reduced *SP3* expression in the *dep1* mutant. It is possible that this reduction is linked to the shorter panicle phenotype observed in both *sp3* and *dep1* knockout mutants. DEP1 interacts with the GRAS transcription factor GRAIN NUMBER ASSOCIATED (GNA) to enhance the inhibitory effect of GNA on *OsCKX2* expression, and GNA is involved in the BR signaling pathway.^[^ ^37^, ^38^ ^]^ In addition, DEP1 interacts with OsMYB86, an R2R3‐MYB transcription factor, positively regulates BR signaling by directly binding to the promoter of its downstream target gene *BRASSINOSTEROID UPREGULATED 1* (*BU1*).^[^ ^39^ ^]^ Recent studies have shown that tissue‐specific activation of *BRASSINOSTEROID‐DEFICIENT DWARF3* (*BRD3*), a BR catabolic gene, plays a key role in promoting panicle branching and increasing grain yield.^[^ ^40^ ^]^ Several *CKX* genes involved in cytokinin degradation such as *OsCKX2*, *OsCKX3*, *OsCKX5*, and *OsCKX9* were significantly upregulated in the *sp3* mutant, which well explained the significantly reduced levels of trans‐zeatin (tZ)‐type cytokinin in the *sp3* mutant.^[^ ^29^ ^]^ These findings suggest that the SP3‐DEP1‐APO2 module probably regulates BR and CK signaling to control panicle architecture. While the integration of hormonal signaling mechanisms still requires further investigation.

Besides the opposite expression changes of *APO2* both in *sp3‐cri* mutants and *dep1‐cri* mutants, the expression levels of *OsWUS*, *IPA1*, and *RCN2* are reduced in *sp3‐cri* mutants but increased in *dep1‐cri* mutants (Figure S3, Supporting Information). Previous studies have shown that the overexpression of *OsWUS*, *IPA1*, and *RCN2* leads to an increase in panicle branches and spikelets.^[^ ^41^, ^42^, ^43^ ^]^ Therefore, SP3 and DEP1 not only antagonistically regulate *APO2* expression but also co‐regulate other genes involved in panicle development, contributing to differences in grain number and branching patterns among *sp3‐cri‐3*, the double mutants *sp3‐cri‐3*/*dep1‐cri‐1*, and *dep1‐cri‐1* mutants.

### 3.2 Interaction of DEP1‐SP3 Determines Alternative Subcellular Localization of DEP1

DEP1 is a type C Gγ subunit which has an N‐terminal Gγ‐like domain and C‐terminal cysteine‐rich domain.^[^ ^34^ ^]^ The type C Gγ subunits of soybean (GmGγ8, GmGγ9, and GmGγ10) are primarily localized to the plasma membrane, where they exhibit distinct punctate fluorescence, particularly in the case of YFP‐GmGγ8.^[^ ^44^ ^]^ Previous studies showed that mutations in conserved cysteine residues can cause specific Gβγ proteins in mammals to delocalize from the cytoplasm or plasma membrane to the nucleus.^[^ ^45^ ^]^ The subcellular localization of DEP1 remains inconsistent in several studies. DEP1 was believed to locate only on the cell membrane,^[^ ^14^ ^]^ both on cell membrane and nucleus^[^ ^13^ ^]^ or only on the cell nucleus.^[^ ^46^ ^]^ A recent study demonstrated that GNA, a protein associated with the BR signaling pathway, promotes the translocation of DEP1 into the nucleus, with GNA overexpression resulting in a marked increase in its nuclear accumulation.^[^ ^37^, ^38^ ^]^ In this study, full‐length DEP1 protein exhibited a punctate pattern and was predominantly enriched at the plasma membrane, similar to GmGγ8 (Figure 3E). And DEP1‐GFP fluorescence signals are observed in the cell membrane and nucleus when SP3 interacts with DEP1 (Figure 3E). Obviously, the DEP1 protein exhibits intracellular mobility and SP3 modulates the cytoplasmic–nuclear shuttle of DEP1. To further explore the underlying mechanism of DEP1 relocalization, biochemical experiments confirmed that SP3 interacts with the C‐terminal of DEP1 rather than with the N‐terminal of DEP1 (Figure S6, Supporting Information). Subcellular localization analyses of truncated DEP1 proteins showed that the C‐terminus is more easily anchored to the plasma membrane than the N‐terminus (Figure S7, Supporting Information). Thus, DEP1 membrane localization property is probably blocked because the C‐terminus of DEP1 is occupied by the interacting protein SP3, resulting in its retention in the nucleus together with SP3. The discovery adds a further dimension to current knowledge that contributes to DEP1 signal transduction study.

### 3.3 A Suggested Working Model of SP3 Regulating Panicle Structure

**Figure 7:**
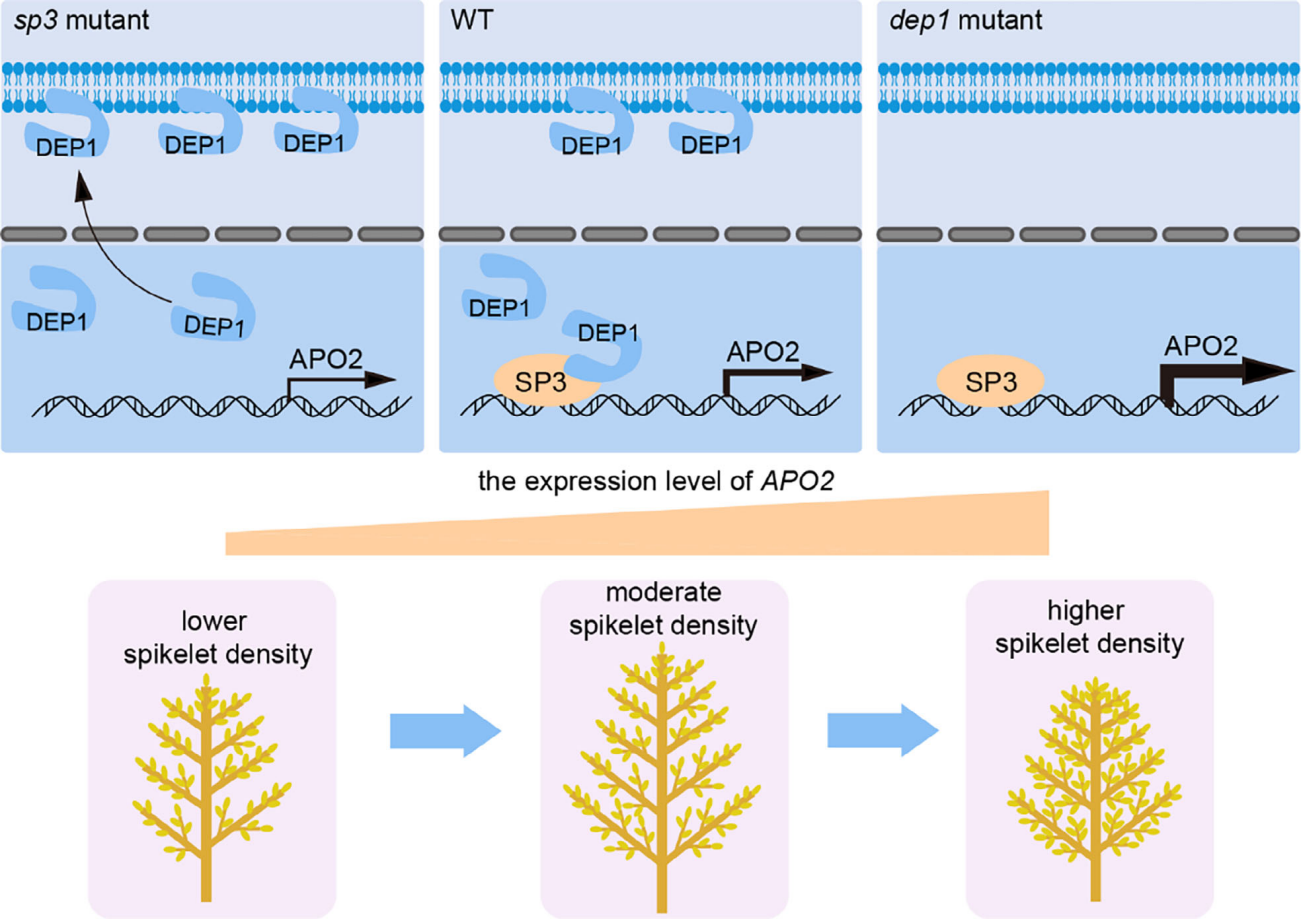
A proposed model for the role of the SP3‐DEP1‐APO2 circuit in regulating rice inflorescence architecture. In WT (center), SP3 interacts with DEP1, promoting its translocation from the plasma membrane to the nucleus and preventing its return to the membrane. DEP1 inhibits SP3‐mediated activation of *APO2*, thereby reducing *APO2* expression. In the *sp3* mutant (left), the absence of SP3 leads to membrane‐localized DEP1 and a marked reduction in *APO2* expression. In the *dep1* mutant (right), the loss of DEP1 abolishes its repressive effect on SP3‐mediated activation of *APO2*, thereby leading to increased *APO2* expression. These regulatory differences lead to moderate branching and spikelet density in WT, reduced branching and spikelet density in the *sp3* mutant, and increased branching and spikelet density in the *dep1* mutant.

Based on these findings, we propose a working model illustrating how SP3 and DEP1 jointly regulate *APO2* expressions to coordinate panicle architecture in rice (**Figure** **7**). DEP1 is located both in the nucleus and plasma membrane due to its nucleus localization domain, N‐terminal Gγ‐like domain and plasma membrane localization domain, C‐terminal cysteine‐rich domain. Since the initiation of young panicle differentiation, the expression level of *SP3* gradually increases. In wildtype plants, SP3 interacts with the C‐terminal cysteine‐rich domain of DEP1 within the nucleus, which inhibits the membrane‐localization capacity of DEP1 and makes more DEP1 locating in nucleus. The DEP1 translocation attenuates the transcriptional activation activity of SP3 on *APO2*, thereby *APO2* expression keeping a moderate level and IM keeping moderate activity. Therefore, the wildtype has panicles with moderate spikelet density.

In the *sp3* mutant, *APO2* expression is downregulated, and IM activity is decreased without the promotion of SP3 and the regulation of the SP3‐DEP1 protein interaction, which results in fewer primary and secondary branches, and lower spikelet density. In the *dep1* mutant, the absence of DEP1 abolishes its interaction with SP3, thereby releasing the repression on SP3‐mediated activation on *APO2*. Consequently, APO2 expressions are upregulated, IM activity is enhanced, which produces more secondary branches and exhibits higher spikelet density in the mutant. Collectively, the SP3‐DEP1‐APO2 module well explains the performance of panicles in the wildtype and mutants and provides a molecular basis for high‐yield rice breeding strategies.

## 4 Experimental Section

### Plant Materials and Phenotypic Evaluations

In this study, Zhonghua 11 (ZH11, *Oryza sativa ssp*.), a japonica variety, was used as the WT and the receptor for genetic transformation. All the materials were grown at the experimental field of Huazhong Agricultural University in summer in Wuhan (30.4°N, 114.2°E), and in winter in Lingshui (110 2 E, 18 30N), during 2021–2024. All transgenic plants in this study were planted at the distance of 16.5 cm between plants within a row and of 25.0 cm between rows. The panicle length, primary branches and secondary branches per panicle, spikelets per panicle and spikelet density were evaluated. Phenotypic measurements were performed on three independent transgenic lines. Significant differences were determined with the Student's T‐test for each set of materials.

### Vector Construction and Genetic Transformation

The knockout vector was constructed using a multitarget CRISPR/Cas9 vector system.^[^ ^47^ ^]^ To generate CRISPR constructs for knocking out of genes *SP3* (LOC_Os03g55610), *DEP1* (LOC_Os09g26999) and *APO2* (LOC_Os04g51000), the target sequence was designed online (http://crispr.hzau.edu.cn/CRISPR2) for each targeting sequence^[^ ^48^ ^]^ and assembled into two sgRNA expression cassettes driven by promoters OsU6a and OsU6b amplified by PCR. Then, these two fragments were ligated into the binary vector pYLCRISPR‐MH using Golden Gate's cloning method.

To generate constructs overexpressing both *SP3* and *APO2*, full length CDS were amplified with corresponding primers and then inserted into the overexpression vector 1301U to generate the vector *SP3*‐OE and *APO2*‐OE. The primers are listed in Table S2 (Supporting Information).

To generated transgenic plants named *SP3‐flag*, the CDS of *SP3* was inserted into pCAMBIA1301U‐flag vector according to the Gibson Assembly method for overexpression of SP3 fused with 3×flag and transformed into ZH11.^[^ ^49^ ^]^ All these constructs were transformed into ZH11 through Agrobacterium tumefaciens mediated transformation.

The double mutants simultaneously knocking out *SP3* and overexpressing *APO2* (*APO2‐OX/sp3‐cri‐2*) were generated through the vector *APO2‐OE* transformed into *sp3‐cri‐2*. The double mutants of *sp3* and *dep1* (*sp3‐cri‐3*/*dep1‐cri‐1*) through hybridization of *sp3‐cri‐3* and *dep1‐cri‐1*. The double mutants of *sp3* and *apo2* (*sp3‐cri‐2*/*apo2‐cri*) were generated through the vector APO2‐CRI transformed into *sp3‐cri‐2*.

### Histological Observation

ZH11 and *sp3‐cri‐3* for sectioning were cultured in a growth chamber with daily cycles of a LD condition (14 h light at 28 °C, 10 h dark at 24 °C). After 30 days of LD condition, the plants subjected to SD condition (10 h light at 28 °C, 14 h dark at 24 °C) and were sampled at 0, 7, 10, and 13 d simultaneously. The SAM or young panicles were peeled off the sample, and were fixed, dehydrated, embedded, sliced and attached to slides as previously reported.^[^ ^7^ ^]^ Sections stained with toluidine blue and images were photographed with a microscope (Nikon, ECLIPSE Ni‐E).

### Yeast Two‐Hybrid Assay

Yeast two‐hybrid assays were performed using the Match maker two‐hybrid system (Clontech, USA). Full‐length CDSs and truncated CDSs of *SP3* and *DEP1* were amplified from cDNA of ZH11 and cloned into *EcoRI* and *BamHI* restriction sites of *pGADT7* and *pGBKT7*. The primers are listed in Table S2 (Supporting Information). Recombinant plasmids were co‐transformed into Saccharomyces cerevisiae strain AH109. Yeast media SD/‐Leu‐Trp (SD/‐LT) and SD/‐Leu‐Trp‐His‐Ade (SD/‐LTHA) were used as permissive and test medium, respectively.

### Luciferase Complementation Imaging (LCI) Assays

The LCI assay was performed in tobacco leaves as previously described.^[^ ^50^ ^]^ The CDS of *DEP1* was cloned into the *pCAMBIA1300‐nLUC* vector to generate nLuc‐DEP1 and the CDS of *SP3* was cloned into *pCAMBIA1300 cLUC* vector to generate cLuc‐SP3. The constructs were transformed into *A. tumefaciens* strain EHA105, and then transformed into 5‐week‐old *Nicotiana benthamiana* leaves by Agrobacterium‐mediated transformation. The plants were cultured at 25 °C for 48 h. Fluorescence was detected using a 5200 Chemiluminescent Imaging System (Tanon Science and Technology) with 1mM potassium luciferin as the substrate. Related primers’ sequences are listed in Table S2 (Supporting Information).

### BiFC Assays

The BiFC assay followed previously reported methods.^[^ ^51^ ^]^ SP3 cDNA was cloned into vector PVYCE containing C‐terminal YFP fragments and DEP1 cDNA was cloned into vector PVYNE containing N‐terminal YFP fragments. The combinations of plasmids were transformed into rice protoplasts and cultured for 12–16 h in the dark. Fluorescence was captured by a confocal microscope (Leica Microsystems). Related primers’ sequences are listed in Table S2 (Supporting Information).

### Co‐IP Assay

The CDS of SP3 and DEP1 were fused with 3×hemagglutinin (HA) and 3×FLAG tag, respectively. The resulting constructs were co transformed into rice protoplasts and the FLAG empty vector PM999‐FLAG was used as the negative control. After an incubation period in the dark for 16 h, the protoplast total proteins were extracted with 600 mL Co‐IP buffer (50 mm Tris‐HCl [pH 7.5], 150 mm NaCl, 10% glycerin, 1 mm EDTA, 0.5% Triton X‐100, 1 mm DTT, 1 mm PMSF, and 1×Protease Inhibitor Cocktail [Roche. CH]) and incubated with 40 µL FLAG beads with gentle up‐and‐down rotation, for 4 h at 4 °C. Then, the beads were washed five times with wash buffer (50 mm Tris‐HCl [pH 7.5], 100 mm NaCl, 10% glycerin, 1 mm EDTA, 0.1% Triton X‐100, 1 mm DTT, 1 mm PMSF, and 1×Protease Inhibitor Cocktail [Roche. CH]). The immunocomplexes were separated by 10% SDS‐PAGE and detected by western blot with anti‐FLAG (Sigma, F1804, USA) and anti‐HA (Abclonal, AE008, China) antibodies.

### Subcellular Localization Analysis

The full‐length or truncated DEP1 CDSs were cloned into a plant transient expression vector (PM999‐GFP) with a N‐terminal GFP fusion. NLS‐RFP was used as nucleus marker consisting of the RFP fused to a nuclear localization signal (NLS). RLCK57‐RFP was used as plasma membrane marker.^[^ ^52^ ^]^ The combinations of plasmids were transformed into rice protoplasts and cultured for 12–16 h in the dark at room temperature. Fluorescence signals of GFP fusion protein were captured by a confocal microscope (Leica Microsystems). Related primers are listed in Table S2 (Supporting Information).

### RNA Extraction and RT‐qPCR Analysis

Total RNA was extracted from fresh leaves and young panicle using TransZol reagent (TransGen, catalogue no. ET101) and quantified using a NanoDrop 2000 system (ND‐2000C, Thermo, USA). Genomic DNA within the samples was removed using DNaseI (Invitrogen), and first‐strand complementary DNA (cDNA) was synthesized with 4 µg of total RNA using Moloney murine leukemia virus reverse transcriptase kit (Invitrogen) according to the manufacturer's instructions. Reverse transcription quantitative PCR (RT‐qPCR) reactions were performed in 384‐well blocks on the Quant‐Studio 6 Flex Real‐Time PCR System (Applied Biosystems, Thermo Fisher Scientific) using FastStart Universal SYBR Green Master Mix (Roche, CH) in a final volume of 10 µL.

The rice ubiquitin gene (Os03g0234200) was used as an internal control. The relative expression was calculated using the 2^−△△CT^ method. The primers used for real‐time PCR are listed in Table S3 (Supporting Information). Three biological replicates and three technical replicates were used for each sample.

### Transcriptional Regulation Assay in Protoplasts

To analyze the transcriptional activity of SP3 and DEP1, the full‐length of SP3 CDS was fused to the DNA‐binding domain of the GAL4 protein (GAL4‐BD).^[^ ^53^ ^]^ driven by the CaMV 35S promoter used as effector vector, and the full‐length of DEP1 CDS was inserted into the plus vector “None” driven by the CaMV 35S promoter. With respect to the reporter construct, the GAL4‐binding site multimerized five times together with a minimal TATA box were placed upstream of the firefly LUC reporter gene.

To validate the direct regulations of SP3 and DEP1 on *APO2*, the full‐length of SP3 and DEP1 CDS were inserted into the effector and plus vector driven by the CaMV 35S promoter, and the promoter sequence of *APO2* and its mutated derivatives were inserted upstream of firefly LUC to generate the reporter constructs. As a reference, the Renilla LUC gene (Promega) under the control of the *AtUbiquitin3* promoter (Ubi: RLUC) was used. The combinations of plasmids were transformed into rice protoplasts isolated from 2 week old green seedlings by polyethylene glycol‐mediated transformation. The two luciferases lysed from the overnight incubated protoplasts were incubated with the Dual Luciferase Reporter Assay System (Promega) according to the manufacturer's instructions, and the luciferase activity was measured using the Infinite M200 System (Tecan, Männedorf, Switzerland). The relative LUC activity was calculated by the ratio of firefly LUC signal values to those of Renilla LUC.

### Yeast One‐Hybrid Assay

Promoter fragments of *APO2* were cloned into the pLacZi2μ vector to generate the p*APO2*: LacZ reporter constructs, and the full‐length of SP3 CDS was cloned into a pJG45AD vector using corresponding primers. The appropriate pairs of pJG45AD and pLacZi2μ vector were then co‐transformed into yeast strain EGY48. The transformants were subsequently grown on SD/−Trp−Ura medium and tested on SD/−Trp−Ura medium containing 40 µg/mL X‐β‐gal (5‐Bromo‐4‐chloro‐3‐indolyl‐β‐D‐galactopyranoside) at 30 °C. Related primers are listed in Table S2 (Supporting Information).

### EMSA

The full‐length ORFs of SP3 were cloned into pGEX‐6p vectors. Glutathione S‐transferase (GST)‐tagged SP3 protein was expressed in *Escherichia coli* BL21 strain (DE3; TransGen Biotech) and then subjected to purification with glutathione sepharose beads (GE Healthcare) which was the same as that used in our previous study.^[^ ^54^ ^]^ Biotin‐labeled and unlabeled single‐stranded DNA oligonucleotides corresponding to the SP3‐binding regions within the promoters of *APO2* were synthesized and renatured to produce biotin‐labeled probes and unlabeled probes, respectively. The indicated proteins and labeled probes or unlabeled probes were performed using a Light Shift Chemiluminescent EMSA Kit (Thermo Fisher Scientific, Waltham, MA, USA) in accordance with the manufacturer's instructions. Related primers are listed in Table S2 (Supporting Information).

### ChIP‐qPCR

Young panicle (<2 mm) tissues of SP3‐flag and wild‐type ZH11 were crosslinked in 1% formaldehyde under vacuum for 10–15 min and quenched with 0.2 m glycine at room temperature. Chromatin was extracted and fragmented to 200–500 bp in length by sonication, and suspension was incubated with antibodies Anti‐FLAG M2 Affinity Gel (A2220; Sigma, St Louis, MO, USA) at 4 °C overnight. The precipitated and input DNA samples were analyzed via quantitative real‐time PCR. Primers for ChIP‐qPCR are listed in Table S3 (Supporting Information).

### Statistical Analysis

Data analysis was carried out by Student's *t*‐test of Microsoft Excel software.

## Conflict of Interest

The authors declare no conflict of interest.

## Supporting information



Supporting Information

## Data Availability

The data that support the findings of this study are available from the corresponding author upon reasonable request.

## References

[1] Y. Du , B. Wu , Y. Xing , Z. Zhang , J. Adv. Res. 2022, 41, 179. 36328747 10.1016/j.jare.2022.01.012 PMC9637487

[2] K. Komatsu , M. Maekawa , S. Ujiie , Y. Satake , I. Furutani , H. Okamoto , K. Shimamoto , J. Kyozuka , Proc. Natl. Acad. Sci. USA 2003, 100, 11765. 13130077 10.1073/pnas.1932414100 PMC208832

[3] M. Komatsu , M. Maekawa , K. Shimamoto , J. Kyozuka , Dev. Biol. 2001, 231, 364. 11237465 10.1006/dbio.2000.9988

[4] M. Komatsu , A. Chujo , Y. Nagato , K. Shimamoto , J. Kyozuka , Development 2003b, 130, 3841. 12835399 10.1242/dev.00564

[5] H. Tabuchi , Y. Zhang , S. Hattori , M. Omae , S. Shimizu‐Sato , T. Oikawa , Q. Qian , M. Nishimura , H. Kitano , H. Xie , X. Fang , H. Yoshida , J. Kyozuka , F. Chen , Y. Sato , Plant Cell 2011, 23, 3276. 21963665 10.1105/tpc.111.088765 PMC3203427

[6] M. Ashikari , H. Sakakibara , S. Lin , T. Yamamoto , T. Takashi , A. Nishimura , E. R. Angeles , Q. Qian , H. Kitano , M. Matsuoka , Science 2005, 309, 741. 15976269 10.1126/science.1113373

[7] B. Wu , J. Meng , H. Liu , D. Mao , H. Yin , Z. Zhang , X. Zhou , B. Zhang , A. Sherif , H. Liu , X. Li , J. Xiao , W. Yan , L. Wang , X. Li , W. Chen , W. Xie , P. Yin , Q. Zhang , Y. Xing , Nat. Genet. 2023, 55, 1381. 37500729 10.1038/s41588-023-01454-3

[8] J. Kyozuka , S. Konishi , K. Nemoto , T. Izawa , K. Shimamoto , Proc. Natl. Acad. Sci. USA 1998, 95, 1979. 9482818 10.1073/pnas.95.5.1979 PMC33826

[9] K. Ikeda‐Kawakatsu , M. Maekawa , T. Izawa , J. Itoh , Y. Nagato , Plant J. 2012, 69, 168. 21910771 10.1111/j.1365-313X.2011.04781.x

[10] Y. Miao , Q. Xun , T. Taji , K. Tanaka , N. Yasuno , C. Ding , J. Kyozuka , Plant Physiol. 2022, 189, 2210. 35556145 10.1093/plphys/kiac216 PMC9342985

[11] X. Huang , Q. Qian , Z. Liu , H. Sun , S. He , D. Luo , G. Xia , C. Chu , J. Li , X. Fu , Nat. Genet. 2009, 41, 494. 19305410 10.1038/ng.352

[12] Q. Liu , R. Han , K. Wu , J. Zhang , Y. Ye , S. Wang , J. Chen , Y. Pan , Q. Li , X. Xu , J. Zhou , D. Tao , Y. Wu , X. Fu , Nat. Commun. 2018, 9, 852. 29487282 10.1038/s41467-018-03047-9 PMC5829230

[13] H. Sun , Q. Qian , K. Wu , J. Luo , S. Wang , C. Zhang , Y. Ma , Q. Liu , X. Huang , Q. Yuan , R. Han , M. Zhao , G. Dong , L. Guo , X. Zhu , Z. Gou , W. Wang , Y. Wu , H. Lin , X. Fu , Nat. Genet. 2014, 46, 652. 24777451 10.1038/ng.2958

[14] S. Sun , L. Wang , H. Mao , L. Shao , X. Li , J. Xiao , Y. Ouyang , Q. Zhang , Nat. Commun. 2018, 9, 851. 29487318 10.1038/s41467-018-03141-y PMC5829277

[15] D. Lijavetzky , P. Carbonero , J. Vicente‐Carbajosa , BMC Evol. Biol. 2003, 3, 17. 12877745 10.1186/1471-2148-3-17 PMC184357

[16] I. Khan , S. Khan , Y. Zhang , J. Zhou , Planta 2021, 253, 101. 33856565 10.1007/s00425-021-03627-y

[17] Z. Wang , D. C. J. Wong , Z. Chen , W. Bai , H. Si , X. Jin , Front. Plant Sci. 2022, 13, 844201. 35668792 10.3389/fpls.2022.844201 PMC9165642

[18] E. L. Waschburger , J. P. C. Filgueiras , A. C. Turchetto‐Zolet , Genet. Mol. Biol. 2024, 46, 20230109. 10.1590/1678-4685-GMB-2023-0109 PMC10842470 38315880

[19] S. Yanagisawa , Plant Cell Physiol. 2004, 45, 386. 15111712 10.1093/pcp/pch055

[20] Y. Shim , B. Kim , Y. Choi , S. H. Cho , Y. Kim , S. H. Kim , Y. Yim , K. Kang , N. C. Paek , Plant J. 2025, 12, 17175. 10.1111/tpj.17175 39615028

[21] Q. Wu , D. Li , D. Li , X. Liu , X. Zhao , X. Li , S. Li , L. Zhu , Front. Plant Sci. 2015, 6, 833. 26500670 10.3389/fpls.2015.00833 PMC4597119

[22] Q. Wu , X. Liu , D. Yin , H. Yuan , Q. Xie , X. Zhao , X. Li , L. Zhu , S. Li , D. Li , BMC Plant Biol. 2017, 17, 166. 29052517 10.1186/s12870-017-1109-0 PMC5649077

[23] Y. Wu , W. Yang , J. Wei , H. Yoon , G. An , Mol. Cells 2017, 40, 178., 28292004 10.14348/molcells.2017.2261 PMC5386955

[24] M. Zhu , Y. Liu , G. Jiao , J. Yu , R. Zhao , A. Lu , W. Zhou , N. Cao , J. Wu , S. Hu , Z. Sheng , X. Wei , F. Zhao , L. Xie , S. Ahmad , Y. Lin , G. Shao , S. Tang , P. Hu , Plant Biotechnol. J. 2024, 22, 1582. 38245899 10.1111/pbi.14288 PMC11123401

[25] Y. Shim , K. Kang , G. An , N. C. Paek , Plant Cell Physiol. 2019, 60, 2065. 31135055 10.1093/pcp/pcz105

[26] Z. Wei , H. Zhang , M. Fang , S. Lin , M. Zhu , Y. Li , L. Jiang , T. Cui , Y. Cui , H. Kui , L. Peng , X. Gou , J. Li , Mol. Plant 2023, 16, 1759. 37742075 10.1016/j.molp.2023.09.011

[27] Y. Fan , H. Chen , B. Wang , D. Li , R. Zhou , W. Lian , G. Shao , X. Wei , W. Wu , Q. Liu , L. Sun , X. Zhan , S. Cheng , Y. Zhang , L. Cao , Plant Physiol. 2024, 196, 1064. 38996044 10.1093/plphys/kiae367

[28] H. Qin , J. Wang , X. Chen , F. Wang , P. Peng , Y. Zhou , Y. Miao , Y. Zhang , Y. Gao , Y. Qi , J. Zhou , R. Huang , New Phytol. 2019, 223, 798. 30924949 10.1111/nph.15824

[29] Y. Huang , X. Bai , M. Luo , Y. Xing , J. Integr. Plant Biol. 2019, 61, 987. 30302902 10.1111/jipb.12729

[30] K. Tsuda , Y. Ito , Y. Sato , N. Kurata , Plant Cell 2011, 23, 4368. 22207572 10.1105/tpc.111.090050 PMC3269871

[31] J. Liu , Q. Mei , C. Yun Xue , Z. Yuan Wang , D. Pin Li , Y. Xin Zhang , Y. Hu Xuan , Plant Biotechnol. J. 2021b, 19, 418. 33098731 10.1111/pbi.13500 PMC7955884

[32] I. Letunic , S. Khedkar , P. Bork , Nucleic Acids Res. 2021, 49, D458. 33104802 10.1093/nar/gkaa937 PMC7778883

[33] F. Taguchi‐Shiobara , Y. Kawagoe , H. Kato , H. Onodera , A. Tagiri , N. Hara , A. Miyao , H. Hirochika , H. Kitano , M. Yano , T. Seiichi , Breed. Sci. 2011, 61, 17.

[34] Q. Xu , M. Zhao , K. Wu , X. Fu , Q. Liu , J. Genet. Genomics 2016, 43, 495. 27520410 10.1016/j.jgg.2016.06.004

[35] W. Zhu , L. Yang , D. Wu , Q. Meng , X. Deng , G. Huang , J. Zhang , X. Chen , C. Ferrandiz , W. Liang , L. Dreni , D. Zhang , New Phytol. 2022, 233, 1682. 34767634 10.1111/nph.17855

[36] L. Huang , K. Hua , R. Xu , D. Zeng , R. Wang , G. Dong , G. Zhang , X. Lu , N. Fang , D. Wang , P. Duan , B. Zhang , Z. Liu , N. Li , Y. Luo , Q. Qian , S. Yao , Y. Li , Plant Cell 2021, 33, 1212. 33693937 10.1093/plcell/koab041

[37] J. Zhang , Q. Lin , X. Wang , J. Shao , Y. Ren , X. Liu , M. Feng , S. Li , Q. Sun , S. Luo , B. Liu , X. Xing , Y. Chang , Z. Cheng , J. Wan , Plant Cell 2024, 37, koae309. 39660553 10.1093/plcell/koae309 PMC11663551

[38] T. Zou , K. Zhang , J. Zhang , S. Liu , J. Liang , J. Liu , J. Zhu , Y. Liang , S. Wang , Q. Deng , H. Liu , J. Jin , P. Li , S. Li , Plant J. 2023, 116, 1766. 37699038 10.1111/tpj.16464

[39] S. Li , Z. Zhao , T. Liu , J. Zhang , X. Xing , M. Feng , X. Liu , S. Luo , K. Dong , J. Wang , Y. Wang , F. Zhang , R. Miao , W. Luo , C. Lei , Y. Ren , S. Zhu , X. Guo , X. Wang , Q. Lin , Z. Cheng , J. Wan , Plant Cell 2025, 37, koaf122. 40398925 10.1093/plcell/koaf122 PMC12124404

[40] X. Zhang , W. Meng , D. Liu , D. Pan , Y. Yang , Z. Chen , X. Ma , W. Yin , M. Niu , N. Dong , J. Liu , W. Shen , Y. Liu , Z. Lu , C. Chu , Q. Qian , M. Zhao , H. Tong , Science 2024, 383, eadk8838. 38452087 10.1126/science.adk8838

[41] K. Miura , M. Ikeda , A. Matsubara , X. J. Song , M. Ito , K. Asano , M. Matsuoka , H. Kitano , M. Ashikari , Nat. Genet. 2010, 42, 545. 20495564 10.1038/ng.592

[42] M. Nakagawa , K. Shimamoto , J. Kyozuka , Plant J. 2002, 29, 743. 12148532 10.1046/j.1365-313x.2002.01255.x

[43] W. Tanaka , Y. Ohmori , T. Ushijima , H. Matsusaka , T. Matsushita , T. Kumamaru , S. Kawano , H. Y. Hirano , Plant Cell 2015, 27, 1173. 25841039 10.1105/tpc.15.00074 PMC4558701

[44] S. R. Choudhury , N. C. Bisht , R. Thompson , O. Todorov , S. Pandey , PLoS One 2011, 6, 23361. 10.1371/journal.pone.0023361 PMC3154445 21853116

[45] T. Kino , T. Kozasa , G. P. Chrousos , Eur. J. Clin. Invest. 2005, 35, 508. 16101671 10.1111/j.1365-2362.2005.01539.x

[46] J. Liu , Q. Mei , C. Yun Xue , Z. Yuan Wang , D. Pin Li , Y. Xin Zhang , Y. Hu Xuan , Plant Biotechnol. J. 2021, 19, 418. 33098731 10.1111/pbi.13500 PMC7955884

[47] X. Ma , Q. Zhang , Q. Zhu , W. Liu , Y. Chen , R. Qiu , B. Wang , Z. Yang , H. Li , Y. Lin , Y. Xie , R. Shen , S. Chen , Z. Wang , Y. Chen , J. Guo , L. Chen , X. Zhao , Z. Dong , Y. G. Liu , Mol. Plant 2015, 8, 1274. 25917172 10.1016/j.molp.2015.04.007

[48] Y. Lei , L. Lu , H. Y. Liu , S. Li , F. Xing , L. L. Chen , Mol. Plant 2014, 7, 1494. 24719468 10.1093/mp/ssu044

[49] D. G. Gibson , L. Young , R. Y. Chuang , J. C. Venter , C. A. Hutchison, 3rd , H. O. Smith , Nat. Methods 2009, 6, 343. 19363495 10.1038/nmeth.1318

[50] H. Chen , Y. Zou , Y. Shang , H. Lin , Y. Wang , R. Cai , X. Tang , J. M. Zhou , Plant Physiol. 2008, 146, 323. 10.1104/pp.107.111740 PMC2245818 18065554

[51] S. K. Kim , H. Y. Park , Y. H. Jang , K. C. Lee , Y. S. Chung , J. H. Lee , J. K. Kim , Planta 2016, 243, 563. 26542958 10.1007/s00425-015-2426-x

[52] Z. Li , Y. Ao , D. Feng , J. Liu , J. Wang , H. B. Wang , B. Liu , Rice 2017, 10, 6. 28220451 10.1186/s12284-017-0145-6 PMC5318303

[53] J. Ma , M. Ptashne , Cell 1987, 48, 847. 3028647 10.1016/0092-8674(87)90081-x

[54] C. Shen , H. Liu , Z. Guan , J. Yan , T. Zheng , W. Yan , C. Wu , Q. Zhang , P. Yin , Y. Xing , Plant Cell 2020, 32, 3469. 32843433 10.1105/tpc.20.00067 PMC7610279

