# Supplementary material for: SP3 and DEP1 Orchestrate Panicle Architecture by Jointly Regulating APO2 Expression in Rice
Source: Adv Sci (Weinh). 2025 Sep 3;12(44):e08230. doi: 10.1002/advs.202508230 (PMC12667520; doi:10.1002/advs.202508230)
Supplement: Supplementary file 1 — Supporting Information [file ADVS-12-e08230-s001.docx]

**Supplementary information**

**
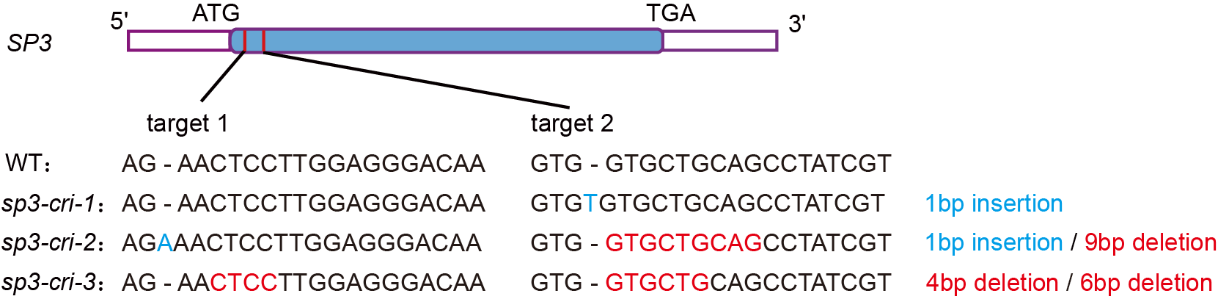
**

**Supplementary Figure 1. The genotypes in three *SP3* knockout mutants.**

The sequence alignment shows mutations in the editing targets. Blue letters represent the insertion sequence and red hyphens represent deletion sequence. The mutant in all lines is homozygous.


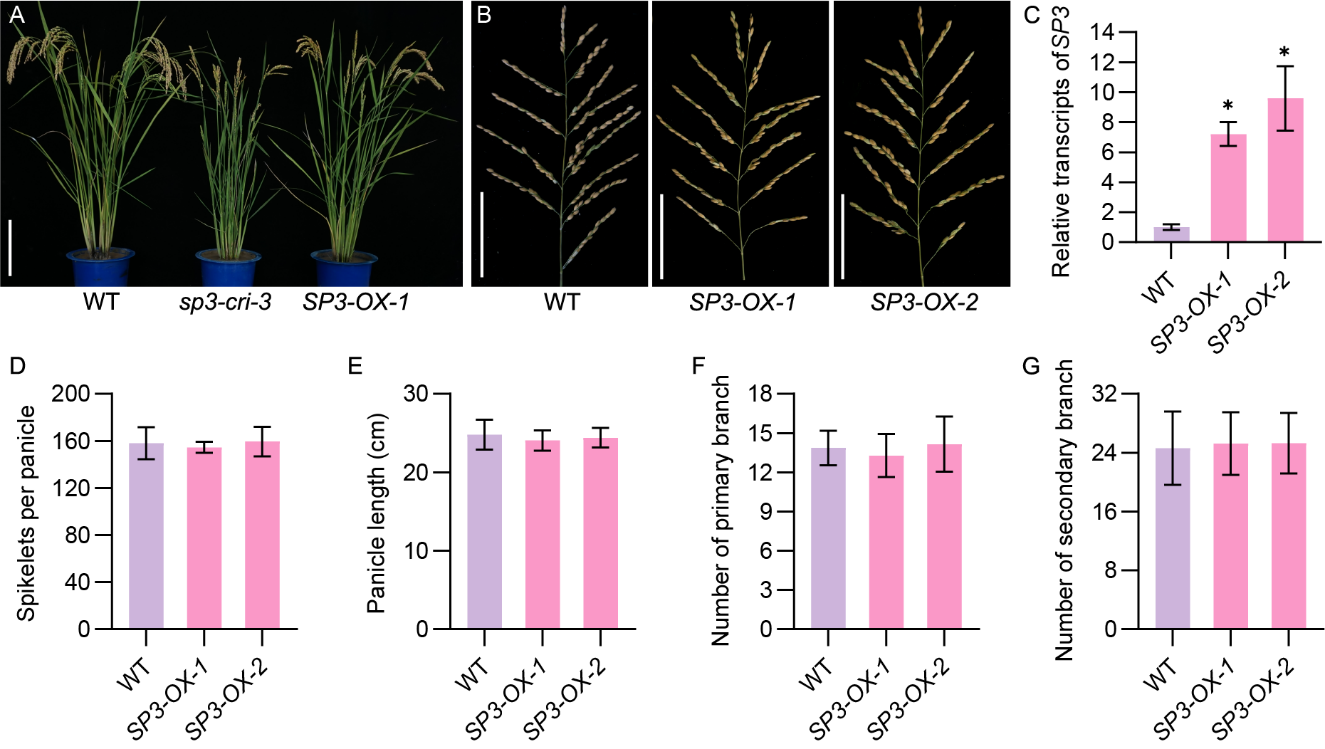


**Supplementary Figure 2 The performance of Overexpression of *SP3***

(A) Comparison of the whole plants at the maturation stage between WT, *sp3-cri-3* and *SP3-OX-1*. Scale bars, 20 cm. (B) Comparisons of the panicle architecture at the maturation stage between WT, *SP3-OX-1* and *SP3-OX-2*. Scale bars, 10 cm. (B) Relative *SP3* expression levels in WT and *SP3-OX*. The data represent the mean ± SD (n=3). *P* values were calculated by two-sided paired Student’s *t*-test, **P* < 0.05. Comparison of spikelets per panicle (C), panicle length (D), number of primary branches (E) and secondary branches (F) among WT and *SP3-OX* (n = 10 panicles). The data represents the mean ± SD (n = 10).


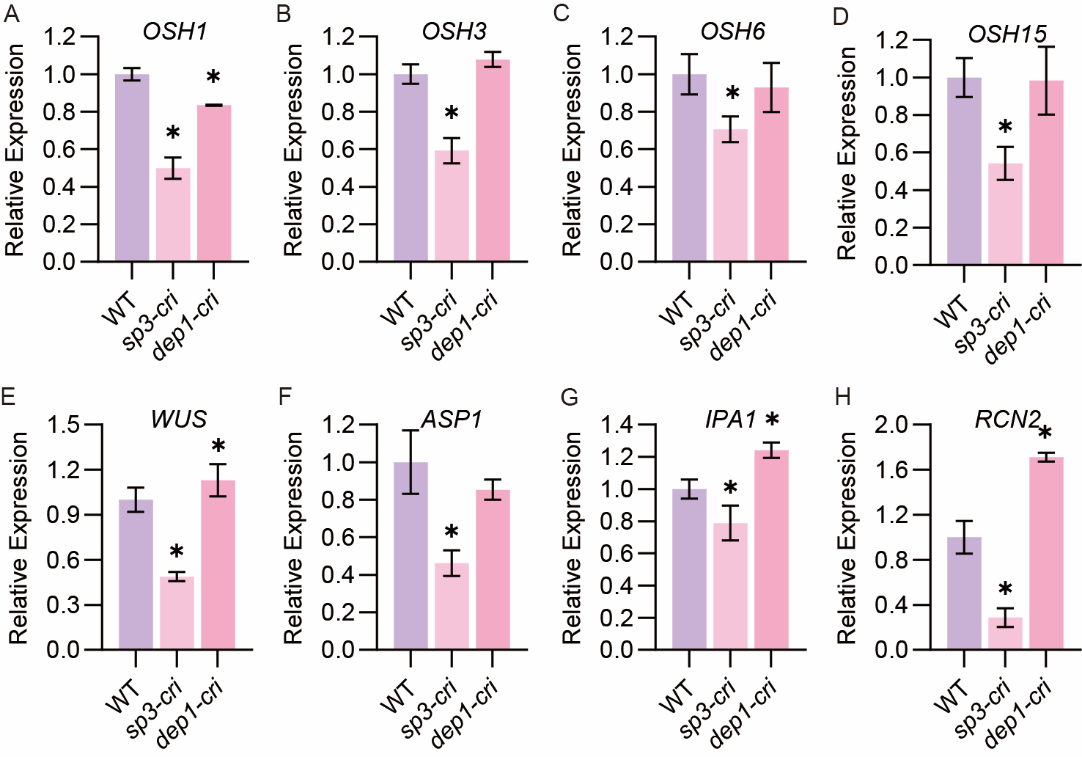


**Supplementary Figure 3. Expression of genes involved in meristem activity and panicle development in WT, *sp3-cri* and *dep1-cri* plants.**

Expression comparison of *OSH1* (A), *OSH3* (B), *OSH6* (C), *OSH15* (D), *OsWUS* (E), *ASP1* (F), *IPA1* (G) and *RCN2* (H) in 2-mm young panicles by RT-qPCR between WT and mutants. Values are given as mean ± SD (n = 3). **P* < 0.05 by Student’s *t* test.


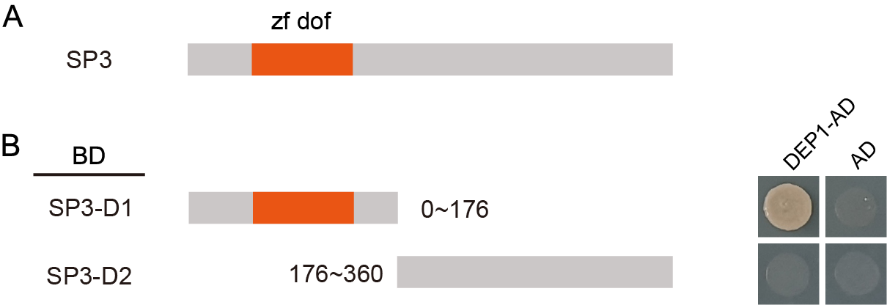


**Supplementary Figure 4 Interactions between truncated SP3 proteins and DEP1**

(A) Functional domain pattern diagram of SP3. The functional domains comprise zf-dof (zinc finger Dof domain). (B) Yeast two-hybrid assays for the interactions between truncated SP3 proteins and DEP1.


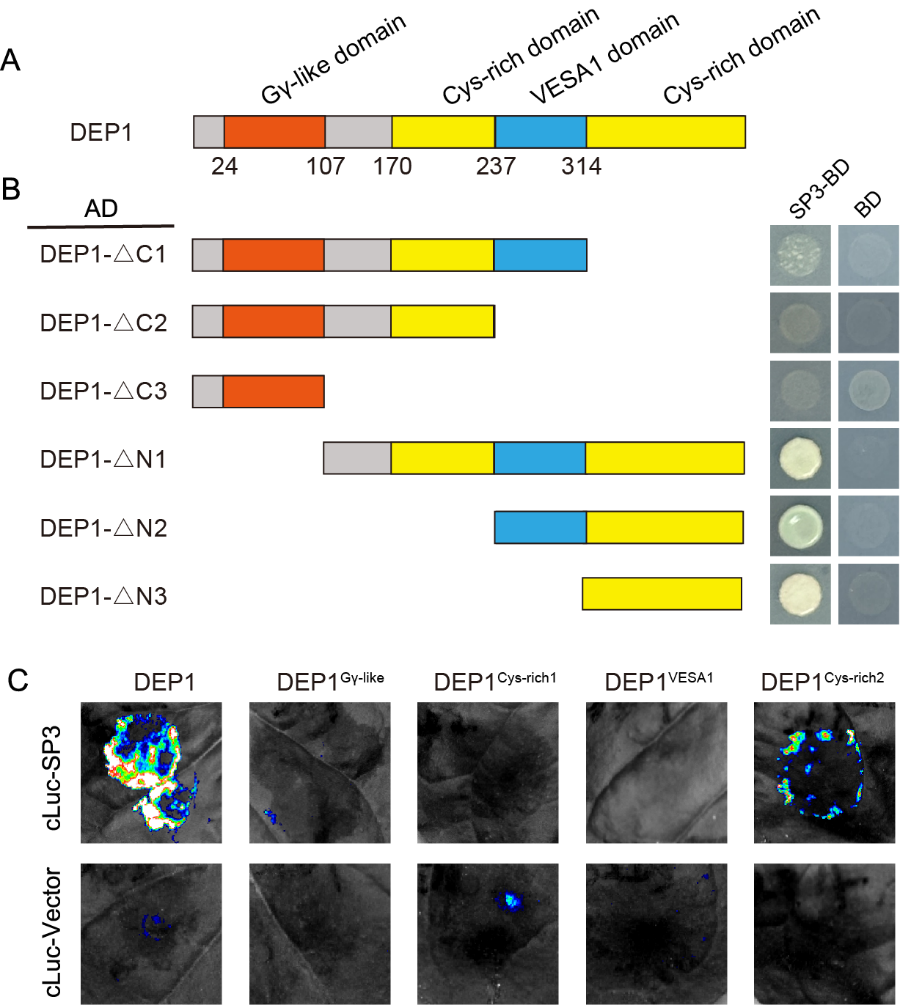


**Supplementary Figure 5 Interactions between SP3 and truncated DEP1 proteins**

(A) Functional domain pattern diagram of DEP1. The functional domains comprise Gγ-like domain, Cys-rich domain and VESA1 (variant erythrocyte surface antigen-1) domain. (B) Yeast two-hybrid assays for the interactions between the truncated DEP1 proteins and SP3. ∆C and ∆N represent several C- and N-terminally truncated DEP1 deletion variants. (C) Luciferase complementation imaging assay showing the interaction between the truncated DEP1 proteins and SP3.


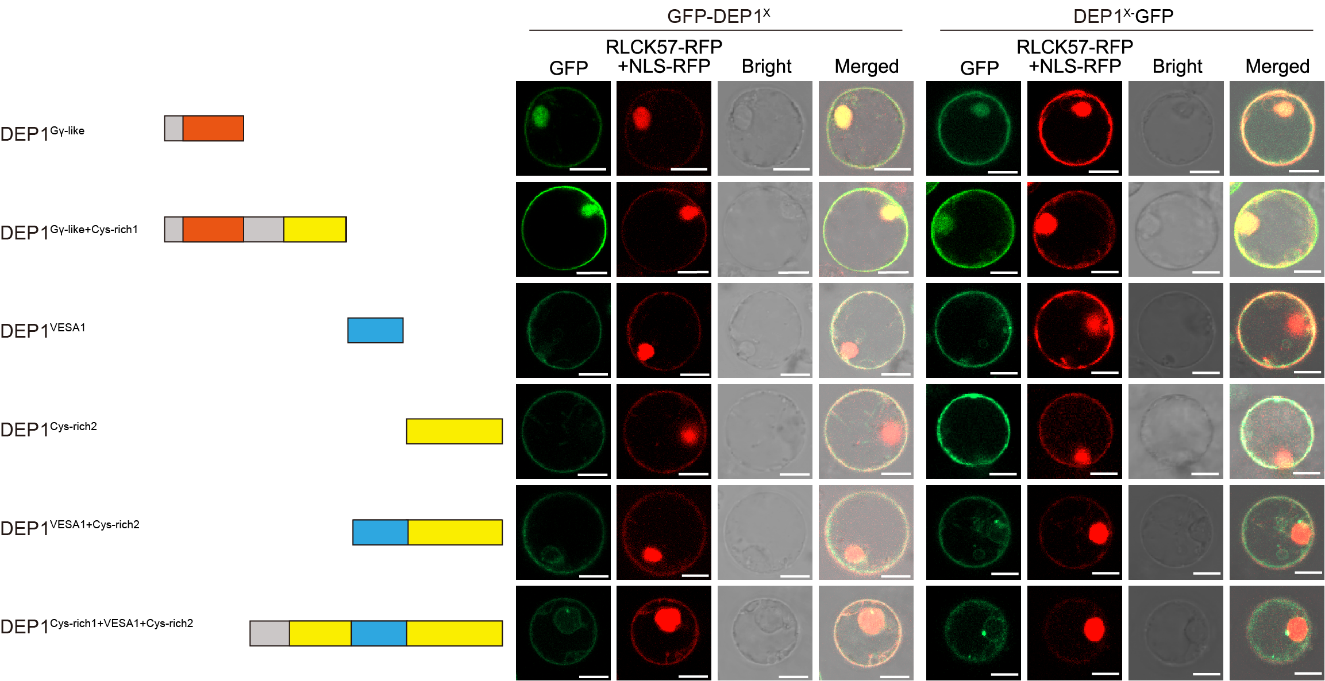


**Supplementary Figure 6 Subcellular localization of the truncated DEP1 proteins**

Analysis of the subcellular localization of DEP1 protein domains using rice protoplast transient expression. Scale bars, 10 μm.


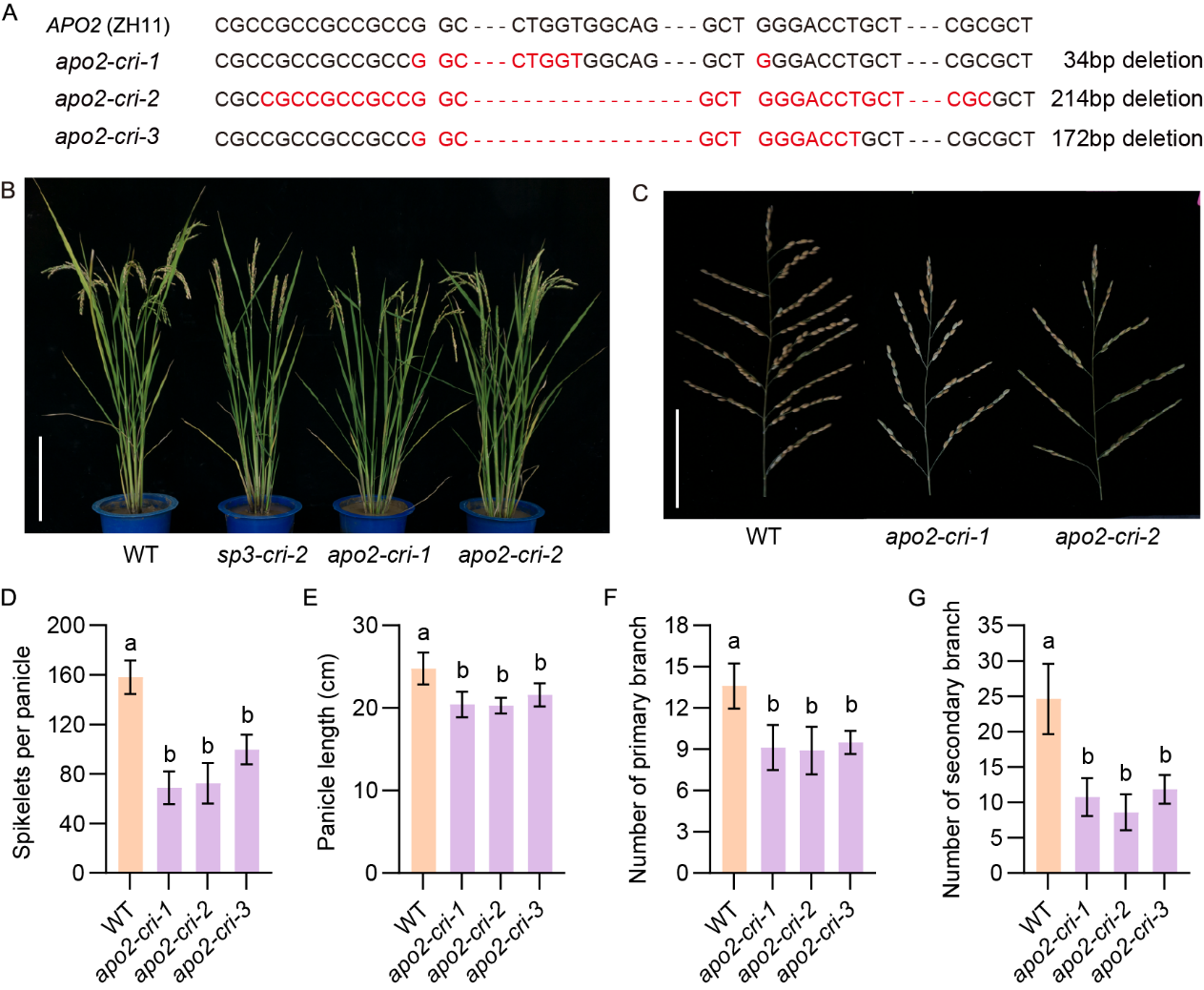


**Supplementary Figure 7 Panicle performance of** ***APO2* knockout mutants**

(A) Mutations in *APO2* knockout mutants. Comparisons of the whole plants (B) and panicle architecture (C) at the maturation stage between WT, *sp3-cri-2* and *APO2* knockout mutants. Scale bars, 20 cm (B), 10 cm (C). Spikelet per panicle (D), Panicle length (E), number of primary branches (F) and secondary branches (G) of WT and *APO2* knockout mutant plants (n = 10 panicles). Values are given as mean ± SD (n = 10). The different lowercase letters above the histogram indicate significant differences by Duncan’s test (*P* < 0.05).


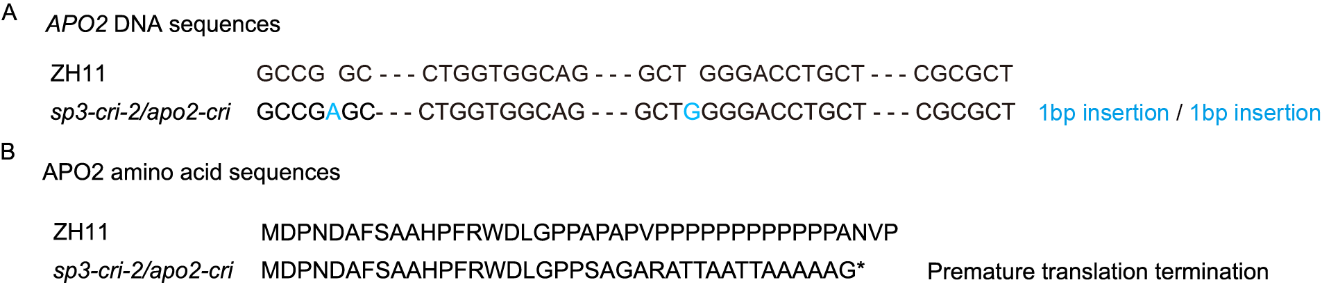


**Supplementary Figure 8 The genotypes of *SP3* and *APO2* double knockout mutants.**

The DNA sequence of *APO2* (A) and amino acid sequence of APO2 (B) in the *SP3* and *APO2* double knockout mutant. Inserted nucleotides are shown in blue. The mutant is homozygous. The *sp3-cri-2*/*apo2-cri* double mutant was generated by transforming the *apo2-cri* construct into the *sp3-cri-2* knockout background.


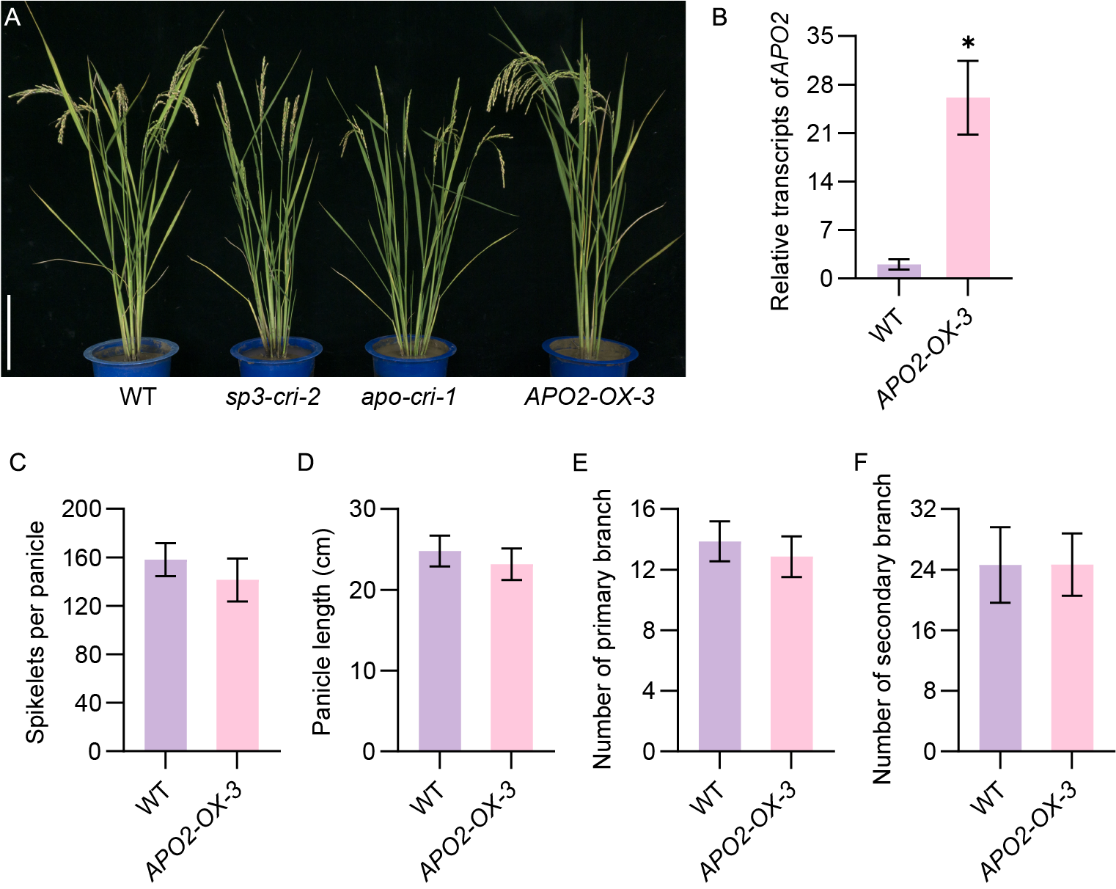


**Supplementary Figure 9 The performance of Overexpression of *APO2***

(A) Comparison of the whole plants at the maturation stage between WT, *sp3-cri-2*, *apo2-cri-1* and *APO2-OX-3*. Scale bars, 20 cm. (B) Relative *APO2* expression levels in WT and *APO2-OX-3*. The data represent the mean ± SD (n=3). *P* values were calculated by two-sided paired Student’s *t*-test, **P* < 0.05. Comparison of spikelet per panicle (C), panicle length (D), number of primary branches (E) and secondary branches (F) among WT and *APO2-OX-3* (n = 10 panicles). The data represents the mean ± SD (n = 10).

**
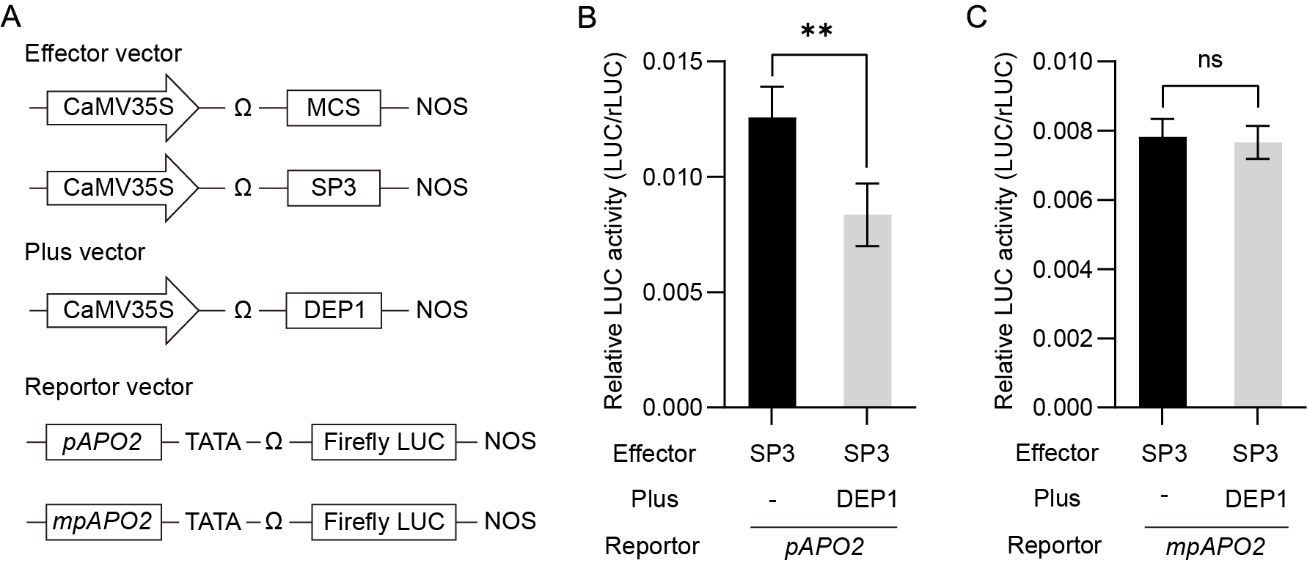
**

**Supplementary Figure 10 DEP1 inhibits the transcriptional activation activity of SP3-induced**

(A) The full-length CDS of SP3 and DEP1 were driven by the CaMV35S promoter. A construct with no gene inserted (None) was used as a negative control. (B-C) Relative LUC activity of combinations of the different effectors and reporters described above. Relative LUC activity is represented by the ratio of signal values of firefly LUC to that of *Renilla* LUC (REN). Data are shown as the mean ± SD of three independent transformants. Different lowercase letters indicate significant differences among the different combinations between reporters and effectors by Duncan’s multiple range tests (*P*<0.05).


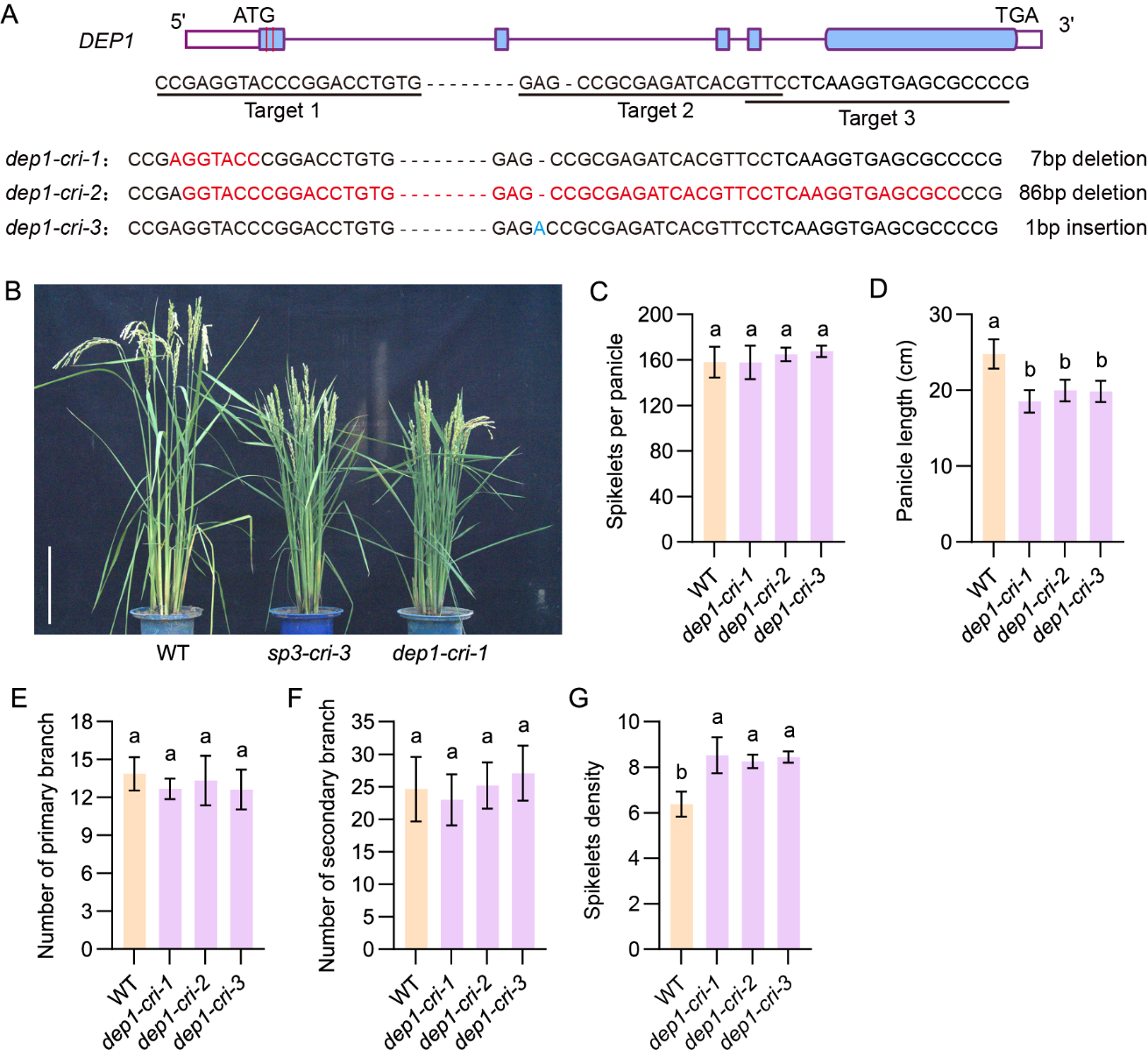


**Supplementary Figure 11 Comparison of panicle traits between *DEP1* knockout mutants and wild type.**

(A) Different mutation types of *DEP1* knockout mutants. (B) Comparisons of the whole plants at the maturation stage between wild-type and *DEP1* knockout mutants. Scale bars, 20 cm. comparison of spikelet per panicle (C), panicle length (D), number of primary branches (E) and secondary branches (F) and spikelet density (G) between WT and *DEP1* knockout mutant plants (n = 10 panicles). Values are given as mean ± SD (n = 10). The different lowercase letters above the histogram indicate significant differences by Duncan’s test (*P* < 0.05).

**Table S1 Amino acid sequences and mutation types of Dof15 in WT and *sp3-cri* mutants**

| Plants | Amino acid sequences | Mutation types |
| --- | --- | --- |
| WT | MIQELLGGTTMDQLKGASALNHASLPVVLQPIVSNPSPTSSSSTSSRSSAQATQQRSSSATSSPHGQGQGGGAAEQAPLRCPRCNSSNTKFCYYNNYNLTQPRHFCKTCRRYWTKGGALRNVPIGGGCRKPRPMPAPVAKPPMSCKAAPPLGLGGGPVSWASGQQAATAHLMALLNSARGVQGHGGSNVHRLLGLDTMGHLQILPGAPNGAGAGTAASLWPQSAPRPVTPPPPHMDSQLGMGTLGHHDVLSSLGLKLPSSASSSPAASYYSDQLHAVVSNAGRPQAPYDVATASLPCTTAVTSLPSALSSVSAAAPTSNTVGMDLPPVSLAAPEMQYWNGPAAMSVPWPDLPTPNGAFP* |  |
| *sp3-cri-1* | MIQELLGGTTMDQLKGASALNHASLPVCAAAYRVQPVAHVVVVDVVALVGAGDAAEVVVGDLVAARAGAGWRRGGAGAAAVPAVQLVEHQVLLLQQLQPHPAAPLLQDVPPVLDQGRRAPQRPHRRRVPQAAPHAGAGRQAAHVLQGRAAARPRRRASVLGLRAAGRHRAPHGAAQQRQGSAGPRRQQCPPASWAGHHGSPPDPARRSQWRRRRHGGVALATVRAAAGHSTAAAHGLPARHGDAGPPRRAVEPRPQAALVGVVLAGGELLQRPAARGGEQRGAPPGAVRRRHRVPPLHHRGDLTPVGAVERLRRRADQQHGRDGPATRVARRAGDAVLEWPGGDVGAVAGLAHSQRRVPMX | loss of function |
| *sp3-cri-2* | MIQETPWRDNHGPAQGRQRSEPRLPAGAYRVQPVAHVVVVDVVALVGAGDAAEVVVGDLVAARAGAGWRRGGAGAAAVPAVQLVEHQVLLLQQLQPHPAAPLLQDVPPVLDQGRRAPQRPHRRRVPQAAPHAGAGRQAAHVLQGRAAARPRRRASVLGLRAAGRHRAPHGAAQQRQGSAGPRRQQCPPASWAGHHGSPPDPARRSQWRRRRHGGVALATVRAAAGHSTAAAHGLPARHGDAGPPRRAVEPRPQAALVGVVLAGGELLQRPAARGGEQRGAPPGAVRRRHRVPPLHHRGDLTPVGAVERLRRRADQQHGRDGPATRVARRAGDAVLEWPGGDVGAVAGLAHSQRRVPMX | loss of function |
| *sp3-cri-3* | MIQELEGQPWTSSRAPAL* | premature translation termination |

**Table S2 Primers used for vector construction in this study.**

| Primer name | SEQUENCE (5' - 3') |
| --- | --- |
| SP3-1301u-F | TACGAACGATAGCCGGTACCATGATCCAAGAACTCCTTGG |
| SP3-1301u-R | TCTTTGTAATCCATGGTACCTGGGAACGCGCCGTTGG |
| APO2-1301u-F | TACGAACGATAGCCGGTACCAATGGATCCCAACGATGCCTT |
| APO2-1301u-R | TCTTTGTAATCCATGGTACCCACGACATTTAGGATTTAGC |
| DEP1-none-F | GTGGATCCCCCGGGCTGCAGGAATTCATGGGGGAGGAGGCGG |
| DEP1-none-R | GTATCGATAAGCTTGATATCGAATTCTCAACATAAGCAACCACTGA |
| SP3-PM999-HA-F | CGATGTTCCAGATTACGCGGGTACCATGATCCAAGAACTCCTTGG |
| SP3-PM999-HA-R | GTTTGAACGATCGGGAATTGGATCCTCATGGGAACGCGCCGTT |
| DEP1-PM999-FLAG-F | CAAAGATGATGATGATAAAGGTACCATGGGGGAGGAGGCGGTGG |
| DEP1-PM999-FLAG-R | GTTTGAACGATCGGGAATTGGATCCTCAACATAAGCAACCACTGA |
| SP3-BD-F | ATCTCAGAGGAGGACCTGCATATGATGATCCAAGAACTCCTTGG |
| SP3-BD-R | CGCTGCAGGTCGACGGATCCTCATGGGAACGCGCCGTT |
| DEP1-AD-F | ATGGAGGCCAGTGAATTCATGGGGGAGGAGGCGGTGG |
| DEP1-AD-△C1-R | CTCGAGCTCGATGGATCCTCACGCGCAGCGGCAGCACT |
| DEP1-AD-△C2-R | CTCGAGCTCGATGGATCCTCAGGCACAGCCCTTGCAGC |
| DEP1-AD-△C3-R | CTCGAGCTCGATGGATCCTCAGCAACAGTAGCAAAGAC |
| DEP1-AD-△N1-F | ATGGAGGCCAGTGAATTCATGAAGTGCTCACCCAAGTG |
| DEP1-AD-△N2-F | ATGGAGGCCAGTGAATTCATGTGTCCAAGCTGTGGATG |
| DEP1-AD-△N3-F | ATGGAGGCCAGTGAATTCATGGACTGCTTCTCCTGCTC |
| DEP1-AD-R | CTCGTGCTCGATGGATCCTCAACATAAGCAACCACTGAG |
| DEP1-NLUC-F | CGGCGCGCCACTAGTGTCGACATGGGGGAGGAGGCG |
| DEP1-NLUC-R | CGCGTACGAGATCTGGTCGACACATAAGCAACCACTGAGACAG |
| SP3-CLUC-F | TACGCGTCCCGGGGCGGTACCATGATCCAAGAACTCCTTGGAGG |
| SP3-CLUC-R | ACGAAAGCTCTGCAGGTCGACTCATGGGAACGCGCCGTT |
| OSH6-NLUC-F | CGGCGCGCCACTAGTGTCGACATGGAGGATCTGTACAGCAT |
| OSH6-NLUC-R | CGCGTACGAGATCTGGTCGACTGGTCCAATTGTGCCAGTGT |
| SP3-pVYCE-F | CGCCACTAGTGGATCCATGATCCAAGAACTCCTTGGAGG |
| SP3-pVYCE-R | GAGCGGTACCCTCGAGTGGGAACGCGCCGTT |
| DEP1-pVYNE-F | CGCCACTAGTGGATCCATGGGGGAGGAGGCGGTGG |
| DEP1-pVYNE-R | GAGCGGTACCCTCGAGACATAAGCAACCACTGAGACAG |
| DEP1-PM999-F | GCAGATCTATCGATTCTAGAATGGGGGAGGAGGCGGTGG |
| DEP1-PM999-△C1-R | TTGCTCACCATGGCTCTAGACGCGCAGCGGCAGCACT |
| DEP1-PM999-△C2-R | TTGCTCACCATGGCTCTAGAGGCACAGCCCTTGCAGC |
| DEP1-PM999-△C3-R | TTGCTCACCATGGCTCTAGATCAGCAACAGTAGCAAAGAC |
| DEP1-PM999-△N1-F | GCAGATCTATCGATTCTAGAATGAAGTGCTCACCCAAGTG |
| DEP1-PM999-△N2-F | GCAGATCTATCGATTCTAGAATGTGTCCAAGCTGTGGATG |
| DEP1-PM999-△N3-F | GCAGATCTATCGATTCTAGAATGGACTGCTTCTCCTGCTC |
| DEP1-PM999-R | TTGCTCACCATGGCTCTAGAACATAAGCAACCACTGAGAC |
| pAPO2-a-LacZ-F | GTACCCGGGGATCTGTCGACCTCGAGTCCTCCAAGTATTGGACAA |
| pAPO2-a-LacZ-R | TATACATACAGAGCACATGCCTCGAGATCGATGTATCTGCATTCAC |
| pAPO2-b-LacZ-F | GTACCCGGGGATCTGTCGACCTCGAGACTCCTATAAGGCTTACTGTG |
| pAPO2-b-LacZ-R | TATACATACAGAGCACATGCCTCGAGTCTAGTAAGCCCTATTACGTGC |
| pAPO2-c-LacZ-F | GTACCCGGGGATCTGTCGACCTCGAGTTGCTACCTCCAAGGAGAG |
| pAPO2-c-LacZ-R | TATACATACAGAGCACATGCCTCGAGTAATGTGGCGCACGTCTCAAG |
| pAPO2-d-LacZ-F | GTACCCGGGGATCTGTCGACCTCGAGAACAGTACGCATGCAGGAGT |
| pAPO2-d-LacZ-R | TATACATACAGAGCACATGCCTCGAGTAGCAACGGCGTCACTGTTC |
| pAPO2-e-LacZ-F | GTACCCGGGGATCTGTCGACCTCGAGTCCAGAATGGTAAAATAATTC |
| pAPO2-e-LacZ-R | TATACATACAGAGCACATGCCTCGAGAATGTTTTTGATTCCGAGGG |
| SP3-pGEX-F | CGGAATTCCCGGGTCGACTCGAG ATGATCCAAGAACTCCTTGG |
| SP3-pGEX-R | TCAGTCACGATGCGGCCGCTCGAGTCATGGGAACGCGCCGTTG |
| Bio-Probe-F | ACGCATGCAGGAGTATCTTTTTTCTTCGGAAGAGGAACAGTGAC |
| Probe-R | GTCACTGTTCCTCTTCCGAAGAAAAAAGATACTCCTGCATGCGT |
| Probe-m1-F | ACGCATGCAGGAGTACCTTTTTTCTTCGGAAGAGGAACAG |
| Probe-m2-F | ACGCATGCAGGAGTATATTTTTTCTTCGGAAGAGGAACAG |
| Probe-m3-F | ACGCATGCAGGAGTATCGTTTTTCTTCGGAAGAGGAACAG |
| Probe-m4-F | ACGCATGCAGGAGTATCTGTTTTCTTCGGAAGAGGAACAG |
| Probe-m5-F | ACGCATGCAGGAGTATCTTGTTTCTTCGGAAGAGGAACAG |
| Probe-m6-F | ACGCATGCAGGAGTATCTTTGTTCTTCGGAAGAGGAACAG |
| pAPO2-190LUC-F | GTAAAACGACGGCCAGTGCCAAGCTTATCCTTGCTCTCATACAACTG |
| pAPO2-190LUC-R | AGGAAGGGTCTTGCAGATCTAAGCTTTCTCCATAGCTCGTTGCATTG |
| mpAPO2-F | AGTATTTTCTTCGGAAGAGGAACAGTGAC |
| mpAPO2-R | TTCCGAAGAAAATACTCCTGCATGCGTAC |

**Table S3 Primers used for RT-qPCR in this study.**

| Primer name | Sequence (5' - 3') |
| --- | --- |
| Ubi-qRT-F | AACCAGCTGAGGCCCAAGA |
| Ubi-qRT-R | ACGATTGATTTAACCAGTCCATGA |
| SP3-qRT-F | GTCCCTCCCTTGCACCAC |
| SP3-qRT-R | CCATTCCAGTACTGCATCTC |
| APO2-qRT-F | AGGTGCAATCCATGGCTAAG |
| APO2-qRT-R | CGCGTAGCAGTGCACGTAGT |
| RCN2-qRT-F | CCTAGTGATCCATACCTGAGGG |
| RCN2-qRT-R | CTCCCAAAAGAGGCATCAGTAG |
| SPL14-qRT-F | CAAGGGTTCCAAGCAGCGTAA |
| SPL14-qRT-R | TGCACCTCATCAAGTGAGAC |
| WUS-qRT-F | GACGGAGCAGATCAAGATCC |
| WUS-qRT-R | GGAACCAGTAGAAGACGTTCTT |
| ASP1-qRT-F | TATCCAATCTCCAGCAAATCGT |
| ASP1-qRT-R | CTAGCTTTGCATCATAGATCGC |
| OSH1-qRT-F | GCTACCTGAGATTGATGCACA |
| OSH1-qRT-R | CCACCTTCTGACTCTCCGA |
| OSH3-qRT-F | ACAGTCTACACACCCTCTCTAT |
| OSH3-qRT-R | GTTAGAATGACGATTTGTGGGG |
| OSH6-qRT-F | GAAGAAAGGGAAGCTACCAAAG |
| OSH6-qRT-R | GCTTATCTTCTTCCGTGGGATA |
| OSH15-qRT-F | GGAATCGACAGGACTAGATCAG |
| OSH15-qRT-R | GGTGAAAACCTTCCATCATGAC |
| APO2-CHIP-1F | AACACAATGTGTGGGTGTAT |
| APO2-CHIP-1R | CTACCGATCGATACTACGTG |
| APO2-CHIP-2F | CGATAATTAAGGGCACAAGG |
| APO2-CHIP-2R | GCAGAAGATTAGCGTGTCAT |
| APO2-CHIP-3F | CGGTGTCTCTCGATACATTA |
| APO2-CHIP-3R | GCAGAAGATTAGCGTGTCAT |
| APO2-CHIP-4F | TGGTATGTGCAAACTAAGAC |
| APO2-CHIP-4R | TAAGGCATAATGATTGTAT |
| APO2-CHIP-5F | TGAATTAATTAATTACTCAC |
| APO2-CHIP-5R | CCTAATCACCATATTAATGA |
